# The B-WICH chromatin-remodelling complex regulates RNA polymerase III transcription by promoting Max-dependent c-Myc binding

**DOI:** 10.1093/nar/gkv312

**Published:** 2015-04-16

**Authors:** Fatemeh Sadeghifar, Stefanie Böhm, Anna Vintermist, Ann-Kristin Östlund Farrants

**Affiliations:** Department of Molecular Biosciences, The Wenner-Gren Institute, Stockholm University, Sweden

## Abstract

The chromatin-remodelling complex B-WICH, comprised of William syndrome transcription factor, the ATPase SNF2h and nuclear myosin, specifically activates RNA polymerase III transcription of the 5S rRNA and 7SL genes. However, the underlying mechanism is unknown. Using high-resolution MN walking we demonstrate here that B-WICH changes the chromatin structure in the vicinity of the 5S rRNA and 7SL RNA genes during RNA polymerase III transcription. The action of B-WICH is required for the binding of the RNA polymerase machinery and the regulatory factors c-Myc at the 5S rRNA and 7SL RNA genes. In addition to the c-Myc binding site at the 5S genes, we have revealed a novel c-Myc and Max binding site in the intergenic spacer of the 5S rDNA. This region also contains a region remodelled by B-WICH. We demonstrate that c-Myc binds to both sites in a Max-dependent way, and thereby activate transcription by acetylating histone H3. The novel binding patterns of c-Myc and Max link transcription of 5S rRNA to the Myc/Max/Mxd network. Since B-WICH acts prior to c-Myc and other factors, we propose a model in which the B-WICH complex is required to maintain an open chromatin structure at these RNA polymerase III genes. This is a prerequisite for the binding of additional regulatory factors.

## INTRODUCTION

Transcription by RNA polymerase III (RNA pol III) in eukaryotic cells is closely associated with cell growth and proliferation. The highly expressed transcripts are short non-coding fragments of RNA that are directly involved in translation, transcriptional elongation and splicing. RNA pol III genes are regulated by nutrients and cellular stress, as well as by proliferative signals generated throughout the cell cycle and differentiation (1,2). Several transcription factors, such as c-Myc and c-Jun, and signalling pathways, such as the MAP kinase pathway, activate these genes (3–5). Tumour suppressors, including the retinoblastoma protein (Rb), p53, ARF and the Maf protein, reduce RNA pol III transcription (6–9). RNA pol III uses specific transcription factors, some of which are gene-specific and used by certain genes due to promoter architecture. TFIIIB, which brings the RNA pol III in to the promoter, is the major target of regulation. Signalling pathways and regulatory proteins either phosphorylate or bind directly to TFIIIB components (2,10,11). Many of the regulators also regulate RNA pol II transcription, but the mechanism is slightly different. For instance, the proliferative regulator c-Myc binds directly to the TFIIIB component Brf1 (or Brf2 on the snU6 RNA genes) (12–16), in a Max-independent manner in RNA pol III transcription. In RNA pol II transcription, however, c-Myc binds to a DNA-binding site, an E-box, as c-Myc-Max dimers at promoter and enhancer regions (17).

Recent genome-wide profiling has shown that the chromatin structure in the vicinity of active RNA pol III genes resembles that of active chromatin at RNA pol II genes (18–20). The histone modification profile at active RNA pol III genes includes histone H3 methylation of lysine 4 (H3K4-me) and acetylation of histone H3 and H4 (18), which are found also at active promoters of RNA pol II genes. The hyperacetylated state is set by several histone acetyl transferase (HATS), two of which, p300 (21) and GCN5 (13), are associated with 5S rRNA genes. In addition, components of RNA pol III factors TFIIIC, TFIIIC200 and TFIIIC90 have intrinsic HAT activity (22,23). The HATs are recruited to genes by transcription factors, such as c-Myc (21–27). On RNA pol III genes, c-Myc recruits the TRRAP complex with GCN5, which in turn acetylates histone H3 (13). Chromatin-remodelling complexes are also associated with RNA pol III transcription. The yeast chromatin-remodelling complex RSC decreases nucleosome density at RNA pol III genes, resulting in a higher RNA pol III occupancy (28,29). In mammalian cells, the chromatin remodeler CHD8 (30) and the histone chaperone FACT (31) are required for RNA pol III transcription, further supporting the idea that chromatin plays a role as transcription regulator. We have previously identified a chromatin-remodelling complex; the ISWI-containing complex B-WICH, which associates with the 5S rRNA genes and 7SL genes and regulates their transcription (32,33). However, the role of the chromatin structure in the regulation of the transcription of the short RNA pol III genes is still poorly understood.

To study the role of chromatin in RNA pol III transcription we have investigated the role of chromatin-remodelling complexes in RNA pol III transcriptionr. We report here investigations into the mechanism by which the B-WICH complex, comprised of WSTF (William Syndrome Transcription Factor), SNF2h and nuclear myosin 1 (NM1), activates RNA pol III transcription. We demonstrate that B-WICH remodels the chromatin locally in the vicinity of the 5S rRNA and 7SL genes, and that this affects transcription. Within the 5S rRDA loci, a region in the intergenic spacer (IGS) is remodelled by B-WICH, and this site is required for the acetylation of histone H3. The remodelled region exposes a DNA-binding site, an E-box, for c-Myc-Max dimers. Binding of Myc-Max is part of a wide gene regulatory network, in which the action of c-Myc is repressed by the alternative interaction of Max with Mxd1 at promoters. The finding that B-WICH is required for the binding of c-Myc-Max to the 5SrDNA locus connects RNA pol III transcription to the Myc/Max/Mxd network that operates on RNA pol II genes (17). Surprisingly, c-Myc also binds to the TFIIIB at the promoter in a Max-dependent manner. These two sites are required for the proper acetylation of histones and most likely for the activation of transcription. We propose a model in which the chromatin structure in the vicinity of the RNA pol III genes imposes a further regulatory level, and in which chromatin-remodelling complexes associating at the genes are required for changing the chromatin structure, thus permitting the binding of regulatory factors.

## MATERIALS AND METHODS

### Cell cultures and treatments

HeLa cells were grown in DMEM high-glucose supplemented with 10% fetal bovine serum (FBS), 50 units/ml penicillin and 50 μg/ml streptomycin. Cells were treated with 75 μM 10058-F4 (Sigma), dissolved in DMSO, for 16 h.

### SiRNA and transfections

HeLa cells were transfected at 50% confluence with WSTF siRNA, c-Myc siRNA, Max siRNA, Brf1 siRNA or scrambled siRNA (sequences are given in the Supplementary Information, Table S1). SiRNA was delivered using lipofectamin RNAi Max (Invitrogen) and the cells were harvested 30–40 h after transfection start, as stated in the text. c-Myc expression vector was delivered using Lipofectamin 2000 (Invitrogen).

### ChIP assays

ChIP was performed as previously described (34). Immunoprecipitated DNA was quantified by polymerase chain reaction (PCR) using the Syber Green mix (Kapa) and all precipitations, except for those performed on histones and histone modifications, were compared to signals at the H27 site in the 45S rDNA (35) or to the actin promoter. The primer pairs used are given in the Supplementary Information (Supplementary Tables S2 and S3). Antibodies used were WSTF (Abcam), SNF2h (Abcam), RNA pol III (kind gift from Dr. Henry and from Abcam), TFIIIA (Abcam), TFIIIC220C (Santa Cruz and Abcam), Brf1 (Abnova and Abcam), GCN5 (Abcam), PCAF (Abcam), p300 (Abnova), TRRAP (Santa Cruz), c-Myc (Santa Cruz and Abcam), Max (Santa Cruz and Abcam), RNA pol II CTD (Abcam), H3-Ac, H3K9-Ac, H3K14-Ac, H3K27-Ac, H3K4-me3, H3K9-me3, H3K27-me3, H4, H4K8-Ac and H4K16-Ac (Abcam).

### High-resolution MNase assay

Cells were cross-linked with 1% formaldehyde for 20 min, and chromatin was prepared as for ChIP, but washed with a buffer containing 25% glycerol, 5 mM magnesium acetate, 50 mM Tris at pH 8.0, 0.1 mM EDTA and 5 mM DTT, as described in Petesch and Lis (36) and modified as described in Vintermist et al. (35). The chromatin was of the same concentration, measured by OD_280_, and the amount of MNase was titrated in each experiment. The digested DNA was evaluated by qPCR, and analysed by calculating ΔCt between the reaction with no MNase and the digested reaction at each concentration. The primer pairs used are given in the Supplementary Information (Supplementary Tables S2 (5S rDNA) and S3 (7SL RNA gene 1).

### Cell extracts and immunoprecipitations

Cell extracts were prepared from nuclei in an extraction buffer containing 0.7 M KCl, 0.05% NP40, 1 mM EDTA and 10 mM Hepes at pH 7.6 (32). Four hundred micrograms of protein was used in each IP. The antibodies used were: WSTF (Abcam), RNA pol III (kind gift from Dr. Henry and Abcam), TFIIIA (Abcam), Brf1 (Abcam), TFIIIC220 (Santa Cruz) and IgG (Abcam).

### Immunoblotting

Immunoblotting was performed using a PVDF membrane (Millipore).

### RNA preparations

RNA was prepared using Qiazol (Qiagen) from cells following the manufacturer's manual. The RNA preparations were subjected to DNAse (1 U for 30 min), and converted to cDNA (Superscript III, Invitrogen) using random primers according to the manufacturer's instructions.

### Protein determination

Protein concentrations were determined using the Bradford reagent (Bio-Rad).

## RESULTS

### The B-WICH complex is required for RNA polymerase III factors to associate with 5S rRNA and 7SL RNA genes

**Figure 1.**
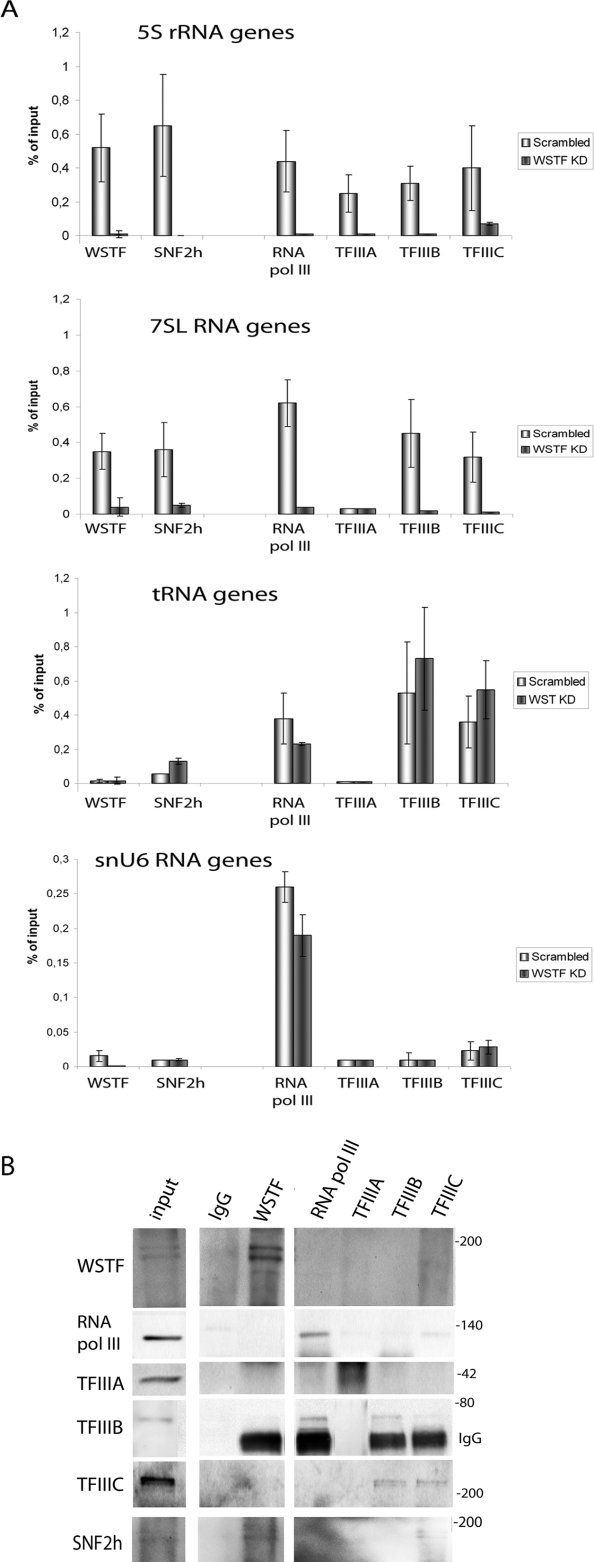
B-WICH associates specifically with the 5S rRNA genes and 7SL RNA genes. **(A)** qPCR of ChIP analyses of WSTF, SNF2h, the RNA pol III and the auxiliary factors TFIIIA, TFIIIB (Brf1) and TFIIIC (TFC220) at the internal promoter of the 5S rRNA, 7SL RNA, tRNA and snU6 genes in cells transfected with scrambled siRNA (Scrambled) and cells transfected with WSTF siRNA (WSTF KD). The results presented are the means of four independent experiments, and the signals are presented as percentage of input signal. The error bars show standard deviations. **(B)** Immunoblotting of a WSTF immunoprecipitation performed on HeLa cell nuclear extract (0.7 M KCl) and the proteins subsequently separated on a 7% SDS-PAGE. The antibodies used to detect interacting proteins are shown on the left. The molecular weights (in kDa) are marked to the right, as is the IgG on the TFIIIB membrane. Input was 10–20% of the IP volume, and IgG was used as a control.

To investigate the underlying mechanism by which the B-WICH complex regulates RNA pol III genes, we first investigated its effect on the binding of components of the RNA pol III transcription machinery. The binding of RNA pol III and its factors, TFIIIA, TFIIIB (Brf1) and TFIIIC (TFIIIC220), to 5S rRNA and the 7SL RNA genes was severely compromised in cells in which the WSTF protein had been knocked down (WSTF KD cells) (Figure 1A). For genes at which no B-WICH, WSTF and SNF2h was found, such as the tRNA genes and the snU6 genes (32), the binding of the RNA pol III factors was not affected (Figure 1A). TFIIIA, a factor specific for 5S rRNA genes, was present only on the 5S rRNA genes. Furthermore, the TFIIIB component Brf1, which is specific for 5S rRNA, tRNA and 7SL RNA genes, was not present on the snU6 genes. In summary, the B-WICH complex is required for the RNA pol III machinery to assemble on the 5S rRNA and 7SL RNA genes. The function is specific to these genes, a finding that supports our earlier work (32) that WSTF knock-down results in a lower level of 5S rRNA and 7SL RNA.

Most regulatory factors of RNA pol III transcription bind directly to the RNA pol III machinery. This led us to investigate whether the WSTF protein interacts directly with RNA pol III and the general RNA pol III transcription factors. However no clear interactions were detected; WSTF antibodies did not precipitate RNA pol III, TFIIIA, TFIIIB-Brf1 or TFIIIC220 (Figure 1B), whereas it precipitated SNF2h. Weak signals of WSTF and SNF2h were detected in the TFIIIC220 precipitate, suggesting weak interactions. Nevertheless, we suggest that the reduction in the association of RNA pol III factors was not a result of WSTF directly being recruited by RNA pol III factors to the 5S rRNA and 7SL RNA genes.

### The B-WICH complex affects the chromatin structure at the 5S rRNA and 7SL RNA genes

**Figure 2.**
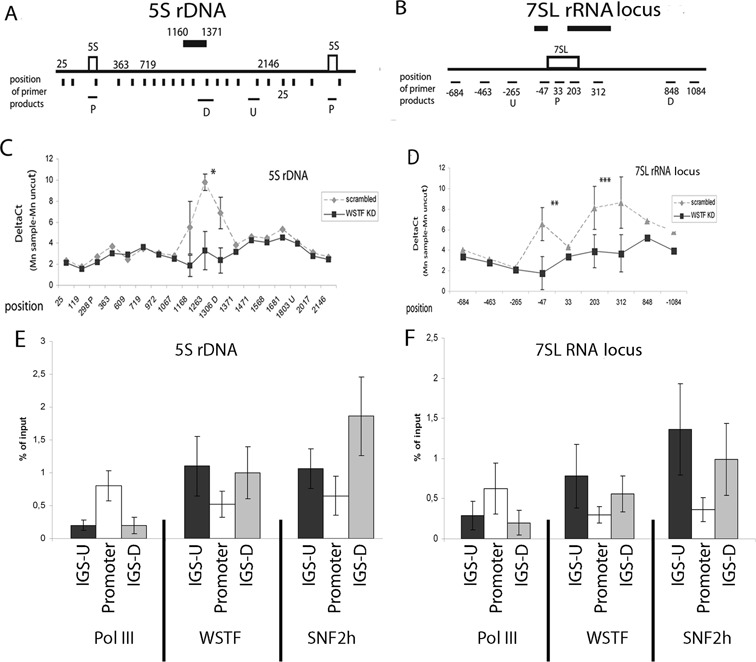
B-WICH remodels localised sites in the IGS of the 5S rDNA and around the 7SL RNA locus. **(A)** Schematic drawing of the 5S rRNA gene repeat, 5S rDNA, showing the positions of the PCR products produced by the primer pairs used, marked as vertical bars under the gene repeat, the gene is marked 5S. The positions are referred to by numbers of the 5S rDNA locus with accession no X12811.1. The positions of primer products from the primer pairs detecting the upstream (U), the promoter (P) and the downstream (D) positions are marked by horizontal bars under the rDNA The effect of WSTF knock-down on chromatin is marked above the gene locus. **(B)** Schematic drawing of the 7SL RNA locus showing positions of the PCR products produced by the primer pairs used. The numbers refer to the position from transcription start site. The gene is marked 7SL, the primers used to detect the positions upstream (U), promoter (P) and downstream (D) are marked under the specific bars. The effects of B-WICH on chromatin are marked above the locus. **(C)** MNase accessibility of the 5S rDNA using cross-linked chromatin from scrambled cells and WSTF KD cells analysed by qPCR using the primer pairs depicted in Figure 2A. The error bars (SD) have been calculated from the results of four independent experiments. (*p = 0.00062, Student's *t*-test, two sample equal variance, at the peak). **(D)** MNase accessibility of the 7SL RNA locus using crosslinked chromatin from scrambled cells and WSTF KD cells analysed by qPCR using the primer pairs depicted in Figure 2B. The error bars (SD) have been calculated from four independent experiments. (**p = 0.01; ***p = 0.0084, Student's *t*-test). **(E)** The distribution of WSTF and SNF2h at the 5S rDNA using the primer pairs detecting the IGS-U = upstream, promoter and IGS-D = downstream. The RNA pol III distribution from the same samples is shown for comparison. The results presented are the means of three independent experiments, and the signals are presented as percentage of input signal. The error bars show standard deviations. **(F)** The distribution of WSTF and SNF2h at the 7SL RNA locus. IGS-U = upstream, promoter and IGS-D = downstream. The RNA pol III distribution from the same samples is shown for comparison. The results presented are the means of four independent experiments, and the signals are presented as percentage of input signal. The error bars show standard deviations.

The reductions in the levels of the RNA pol III factors associated with the 5S rRNA genes and the 7SL RNA genes in WSTF KD cells led us to investigate the effect of B-WICH on the chromatin state at these genes. We used micrococcal nuclease (MNase) on cross-linked chromatin isolated from cells transfected with scrambled siRNA or WSTF siRNA, and analysed the regions around the genes with primer pairs that produced adjoining 100-bp products. The positions of the amplicons are shown in the diagrams of the 5S rDNA and 7SL RNA locus (Figure 2A and B). WSTF knock-down generated a region that was less accessible to MNase in the IGS between the 5S rRNA genes (Figure 2C) (*p* = 0.0006, Student's *t*-test). At the 7SL RNA gene, two positions were less accessible on either side of the gene, both upstream (*p* = 0.025) and downstream (*p* = 0.008), in WSTF KD cells (Figure 2D). The approximate positions of these positions are marked in Figure 2A and B. DNase digestions of permeabilised WSTF KD cells gave similar patterns; WSTF knock-down resulted in a less accessible region downstream of the 5S rRNA genes, and in the vicinity of the 7SL RNA gene (Supplementary Figure S1). In addition, the DNase digestion revealed a region over the 5S rRNA gene that was less accessible in WSTF KD cells, indicating that the B-WICH also here causes a slight change in the chromatin structure.

As the effects of B-WICH were seen outside of the 5S rRNA and at the edges of the 7SL RNA genes, we investigated the association pattern of WSTF and SNF2h in the loci. In contrast to RNA pol III, which associated mainly within the 5S rRNA and 7SL RNA genes, the WSTF and SNF2h signals from ChIPs peaked outside of the genes (Figure 2E and F). Taken together, we conclude that the B-WICH complex remodelled chromatin around the 5S rRNA and 7SL RNA genes from distal positions to the genes.

### B-WICH affects the histone modification patterns at the 5S rRNA and the 7SL genes

**Figure 3.**
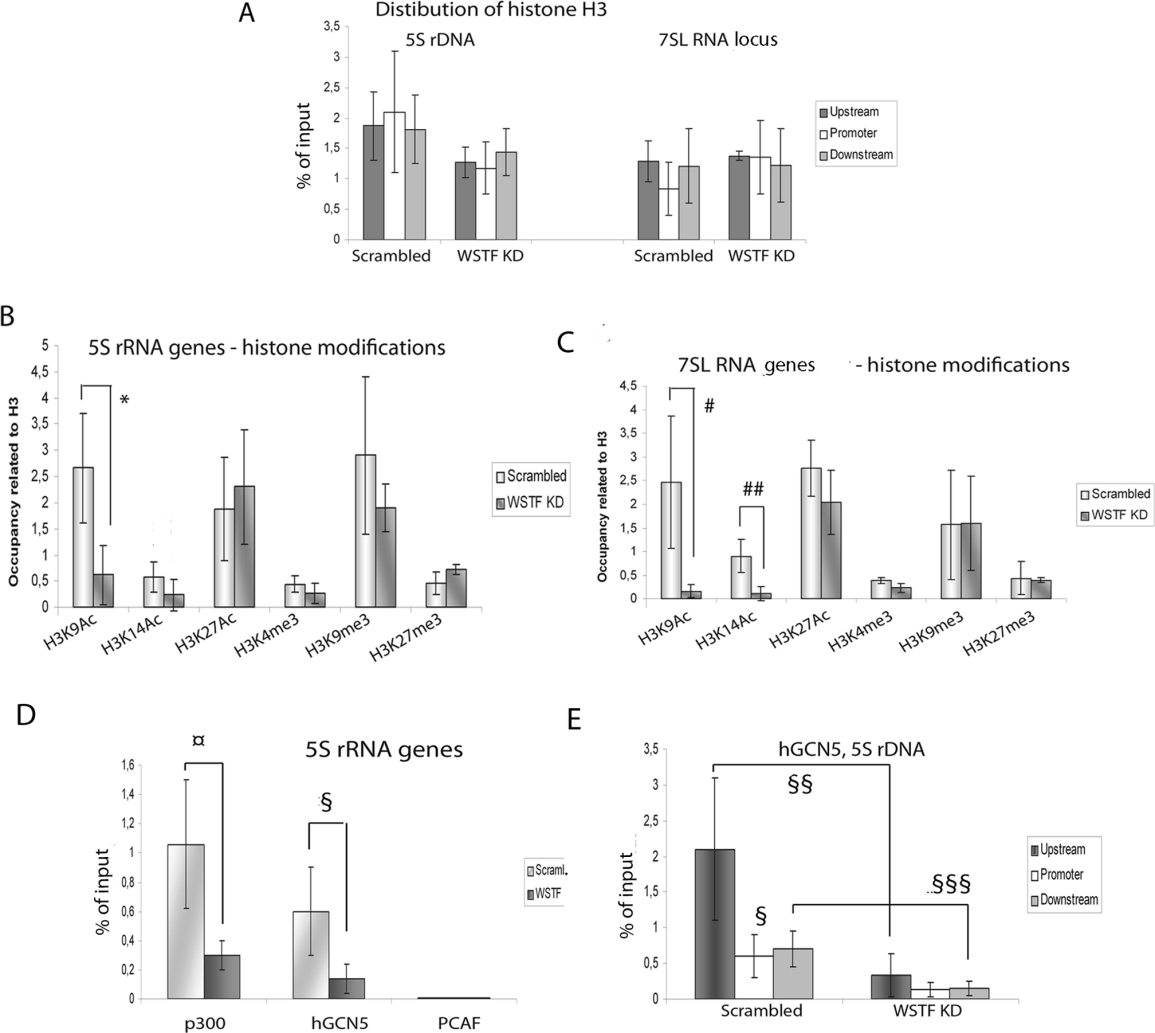
B-WICH induces histone acetylation of H3K9 and H3K14. **(A)** qPCR of ChIP analyses determining the level of histone H3 occupancy at the 5S rRNA and the 7SL RNA genes in scrambled cells and WSTF KD cells. The results presented are the means of four independent experiments, and the signals are presented as percentage of input signal. The error bars show standard deviations. **(B)** qPCR of ChIP analyses determining the occupancy of histone modifications; H3K9-Ac, H3K14-Ac, H3K27-Ac, H3K4-me3, H3K9-me3, H3K27-me3 at the 5S rRNA gene in scrambled and WSTF KD cells at the 5S rRNA gene. The values are adjusted to the level of H3. (*p = 0.0075, Student's *t*-test). The results presented are the means of four independent experiments and the error bars show standard deviations. **(C)** qPCR of ChIP analyses of histone modifications; H3K9-Ac, H3K14-Ac, H3K27-Ac, H3K4-me3, H3K9-me3, H3K27-me3 at the 7SL RNA gene in scrambled and WSTF KD cells. The values are adjusted to the level of H3. (#*p* = 0.015; ## *p* = 0.036, Student's *t*-test) The results presented are the means of four independent experiments, and the error bars show standard deviations. **(D)** qPCR of ChIP analyses of the association of p300, GCN5 and PCAF at the 5S rRNA genes in scrambled cells and WSTF KD cells (¤ *p* = 0.038; § *p* = 0.044, Student's *t*-test). The results presented are the means of four independent experiments, and the signals are presented as percentage of input signal. The error bars show standard deviations. **(E)** Distribution of the binding of GCN5 to the 5S rDNA, using ChIP, in scrambled cells and WSTF KD cells, at upstream, promoter and downstream regions. (§§ *p* = 0.014; §§§ *p* = 0.03, Student's *t*-test). The results presented are the means of four independent experiments, and the signals are presented as percentage of input signal. The error bars show standard deviations.

It is possible that the closed chromatin configuration at the 5S rDNA and 7SL RNA locus upon WSTF knocks down was due to a changed nucleosome density. However, no significant difference in H3 occupancy was observed at the 5S rRNA genes or the 7SL RNA genes in WSTF KD cells compared to that found in cells transfected with scrambled siRNA (Figure 3A). Next, we examined the influence of WSTF knock-down on the activating histone modifications H3K9-Ac, H3K14-Ac, H3K27-Ac and H3K4-me3; and on the modifications associated with silencing, H3K9-me3 and H3K27-me3, at the genes and IGS. WSTF knock-down specifically reduced the levels of H3K9-Ac and H3K14-Ac at different positions at the loci. WSTF knock-down resulted in a reduced level of H3K9-Ac at the 5S rRNA genes, and the levels of both H3K9-Ac and H3K14-Ac at the 7SL RNA genes were affected (Figure 3B and C). The effect on H3 acetylations was also seen outside of the genes; upstream of the 5S RNA gene, WSTF knock-down reduced both H3K9-Ac and H3K14-Ac, while upstream of the 7SL RNA gene the level of H3K9-Ac was reduced in WSTF KD cells (Supplementary Figures S2A and S2B). The level of H3K27-Ac did not change significantly at any of the positions after WSTF knock-down (S2A and S2B), demonstrating that B-WICH influences the histone-acetylation pattern around these two RNA pol III loci.

### The B-WICH complex facilitates the binding of the histone acetyl transferases

To determine the effect of the B-WICH complex on the association of HATS with the 5S rDNA and the 7S RNA locus, we first tested whether binding pattern of different HATs. We found that the p300 and GCN5, but not the PCAF, associate with the 5S RNA genes (Figure 3D), while all three associated with the 7 RNA SL gene (Supplementary Figure S2C). WSTF knock-down resulted in less binding of p300 and hGCN5 at the 5S rRNA genes (Figure 3D), and at the 7 SL RNA gene the binding of all three HATs was reduced (Supplementary Figure S2C).

Since p300 has been reported not to function as a HAT at the 5S rRNA genes (21), we focused on the association pattern of GCN5. GCN5 bound at and around the 5S RNA gene (Figure 3E) and the 7SL RNA gene (Supplementary Figure S2D), and the binding was compromised at all the locations tested in WSTF KD cells (Figure 3E and Supplementary Figure S2D). On the other hand, the binding of GCN5 at the tRNA genes and snU6 genes was not affected by WSTF KD (Supplementary Figure S2E). The GCN5 is recruited to RNA pol III genes as part of the TRRAP complex (13), and also the association of the TRRAP protein with the 5S rRNA and 7SL genes was reduced in WSTF KD cells (Supplementary Figure S4F). We conclude that the action of B-WICH on the 5S rDNA and the 7SL RNA genes resembles that observed on RNA pol I genes, where the recruitment of H3-HATs was compromised, and the level of H3K9-Ac was subsequently affected upon WSTF knock-down (35).

### The B-WICH complex regulates the binding of c-Myc to 5S rRNA and 7SL RNA genes

**Figure 4.**
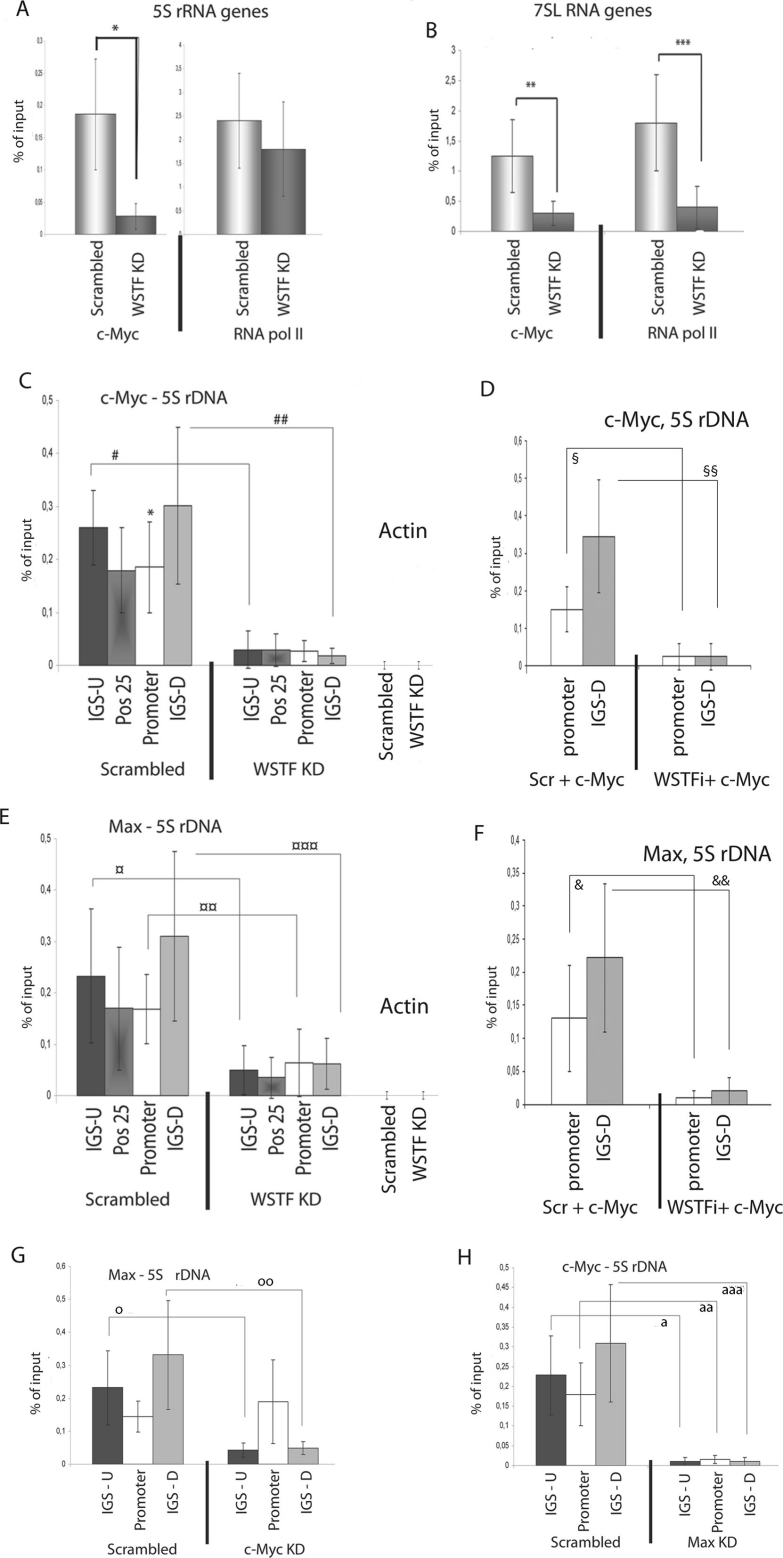
c-Myc associates with the whole 5S rDNA and the 7SL RNA locus. **(A)** The association of c-Myc and RNA pol II with the 5S rRNA genes, detected by qPCR of ChIPs, in scrambled and WSTF KD cells. The error bars (standard deviations) are from of four independent experiments (*p = 0.002, Student's *t*-test). **(B)** The binding of c-Myc and RNA pol II to the 7SL RNA gene, detected by qPCR of ChIPs, in scrambled and WSTF KD cells. The error bars (standard deviations.) are from of four independent experiments (**p = 0.011; ***p = 0.017, Student's *t*-test). **(C)** Distribution of c-Myc at the 5S rDNA in scrambled and WSTF KD cells. The marking of the primer products refers to the positions depicted in Figure 2A and includes the upstream (IGS-U), the promoter and downstream (IGS-D), and the number (Pos 25) to the number in Figure 2A. Actin refers to the promoter of the actin gene. The error bars (standard deviations.) are from four independent experiments (# *p* = 0.007; ## *p* = 0.002, Student's *t*-test). **(D)** The presence of c-Myc at the gene at the IGS-D in cells in which WSTF has been knocked down and c-Myc expressed exogenously for 12 h compared to cells transfected with scrambled controls overexpressing c-Myc. The error bars (standard deviations) are from three independent experiments (§ *p* = 0.014; §§ *p* = 0.026, Student's *t*-test). **(E)** Distribution of the association of Max at the 5S rDNA locus by ChIP in scrambled and WSTF KD cells. The primer pairs detect the upstream (IGS-U), the position 25 in Figure 2A (Pos25), the promoter and the downstream (IGS-D) positions. The error bars (standard deviations) are from three independent experiments (¤ *p* = 0.04; ¤¤ *p* = 0.03; ¤¤¤ *p* = 0.015, Student's *t*-test)**. (F)** The occupancy of c-Myc at the internal promoter of the 5S rRNA gene and in the IGS (IGS-D) in cells in which WSTF has been knocked down and c-Myc expressed exogenously for 12 h compared to cells transfected with scrambled siRNA and overexpressing c-Myc. The error bars (standard deviations.) are from three independent experiments (& *p* = 0.0009; && *p* = 0.0006, Student's *t*-test). **(G)** qPCR analyses of ChIP experiments estimating the association of Max to the 5S rDNA using primer pairs detecting the upstream (IGS-U), the promoter and the downstream (IGS-D) positions. The error bars represent standard deviations from four independent experiments (o *p* = 0.03; oo *p* = 0.003). **(H)** qPCR analyses of ChIP experiments of the distribution of the c-Myc in scrambled and Max KD cells at the 5S rDNA. The positions detected were analysed by the primer pairs giving the products marked IGS-U (upstream), promoter and IGS-D (downstream) in Figure 2A. The error bars represent standard deviations from three independent experiments (a *p* = 0.003; aa *p* = 0.011; aaa *p* = 0.005).

Several factors such as c-Myc and RNA pol II have been suggested to regulate the chromatin structure at RNA pol III genes (13,18–20,37–39). We found both of these factors associated at the 5S rRNA genes, 7SL RNA genes, snU6 genes and tRNA genes (Figure 4A, B and Supplementary Figure S3A). The silencing of WSTF by siRNA, however, reduced the binding of c-Myc only at the 5S rRNA and 7SL RNA genes, and not at the snU6 or tRNA genes (Figure 4A, B and Supplementary Figure S3A). The binding of RNA pol II was compromised only at the 7SL RNA gene in WSTF KD cells (Figure 4B).

Genome-wide studies have revealed that c-Myc binds in a broad peak over RNA pol III genes (37). We investigated the distribution of c-Myc at the 5S rDNA and detected a broad peak. In addition to the association at the gene, c-Myc associated in the IGS between the 5S rRNA genes, with a high level of binding detected using primers amplifying a region approximately 1 kb downstream of the gene (Figure 4C). WSTF knock-down reduced the binding of c-Myc at all positions; around the gene and in the IGS (Figure 4C). The association pattern of c-Myc in the vicinity of the 7SL gene was also broad and the association was severely compromised at all positions in WSTF KD cells (Supplementary Figure S3B). As a control, we measured the binding of c-Myc at the actin gene promoter and detected no binding at this site (4C).

The protein level of c-Myc was lower in WSTF knock-down cells, a finding that was also reflected in the binding of c-Myc to the chromatin factor (Supplementary Figure S3C). In order to ensure that the reduced binding upon WSTF knock-down at the 5S rRNA and 7SL RNA genes was not a result of the lower c-Myc level, we overexpressed c-Myc in cells in which WSTF had been knocked down by siRNA. Overexpression of c-Myc did not result in large increases of the 5S rRNA, 7SL RNA and tRNA, while a large increase was observed in the snU6 RNA level (Supplementary Figure S3D). ChIP experiments investigating the binding of c-Myc in WSTF KD cells overexpressing c-Myc showed that the binding at the genes and in the IGS was reduced at the 5S rDNA (Figure 4D) and the 7SL RNA locus (Supplementary Figure S3E). The binding of c-Myc at the tRNA was retained, although at lower levels (Supplementary Figure S3F). Despite the increase in snU6 RNA, no change in the binding of c-Myc was detected at the snU6 gene (S3F). The finding that the binding of c-Myc at the 5S rDNA and 7SL RNA locus is severely reduced in WSTF KD cells overexpressing c-Myc demonstrates that B-WICH chromatin remodelling is required for c-Myc binding at these loci.

### The B-WICH complex allows c-Myc to bind to a site in the IGS

The peak of c-Myc observed in the IGS of the 5S rDNA and the 7SL RNA locus prompted us to examine the region for E-boxes (CACGTG), to which c-Myc binds as a dimer with Max (17). Several E-boxes, both canonical and non-canonical, are present in the 5S rDNA repeat and in the vicinity of the 7SL RNA genes (schematically depicted in Supplementary Figures S4A and S4B). An E-box is located at position 1192 in the 5S rDNA repeat, which coincides with the region that was protected in WSTF KD cells. This led us to investigate the association of the Max protein with the 5S rDNA locus. We found that Max associated with the 5S rDNA in a pattern similar to that of c-Myc (Figure 4E). No binding was detected at the actin gene promoter (Figure 4E). Similar to c-Myc, the binding of Max was also reduced at all positions in the 5S rDNA in WSTF KD cells. The binding pattern of Max was also compromised in WSTF KD cells in which c-Myc was overexpressed (Figure 4F). Furthermore, Max associated with the 7SL RNA locus in a WSTF dependent manner (Supplementary Figure S4C) and the same pattern was observed in WSTF KD cells overexpressing c-Myc (Supplementary Figure S4D). The binding of Max to the tRNA gene was not affected by WSTF knock-down or the simultaneous expression of c-Myc (S4D), while no Max was detected at the snU6 genes (Supplementary Figure S4E and SF). Taken together, these results demonstrate that B-WICH is required to open chromatin at the 5S rDNA and the 7SL RNA locus to allow for the binding of c-Myc-Max. This suggests that c-Myc functions not only by interacting with Brf1 at the gene, but also by binding as a dimer with Max to the IGS in a similar manner to the binding at RNA pol II genes.

Interestingly, we detected Max associated with the 5S rRNA gene (Figure 4E), suggesting that the c-Myc-Max complex also binds at this position. To elucidate the pattern of interactions between c-Myc and Max, we knocked down c-Myc and Max and studied the association patterns at the 5S rDNA and 7SL RNA locus. c-Myc knock-down resulted in reduced binding of Max in the IGS of the 5S rDNA, while it remained at the gene (Figure 4G), suggesting that Max binds to the gene. The binding of Max did not require c-Myc, further suggesting that also the binding site at the gene is part of the c-Myc/Max/Mxd network. In contrast, Max knock-down abolished the binding of c-Myc at all position tested (Figure 4H). Max also associated with the 7SL RNA locus and c-Myc knock-down reduced the binding outside of the gene, whereas the binding at the gene was less affected (Supplementary Figure S5A). Max associated with tRNA genes and its binding remained in c-Myc knock-down cells (Supplementary Figure S5B). No binding of Max was detected at the snU6 gene (Supplementary Figure S5B)

### c-Myc regulates histone acetylation and RNA pol III recruitment to the 5S rRNA genes

**Figure 5.**
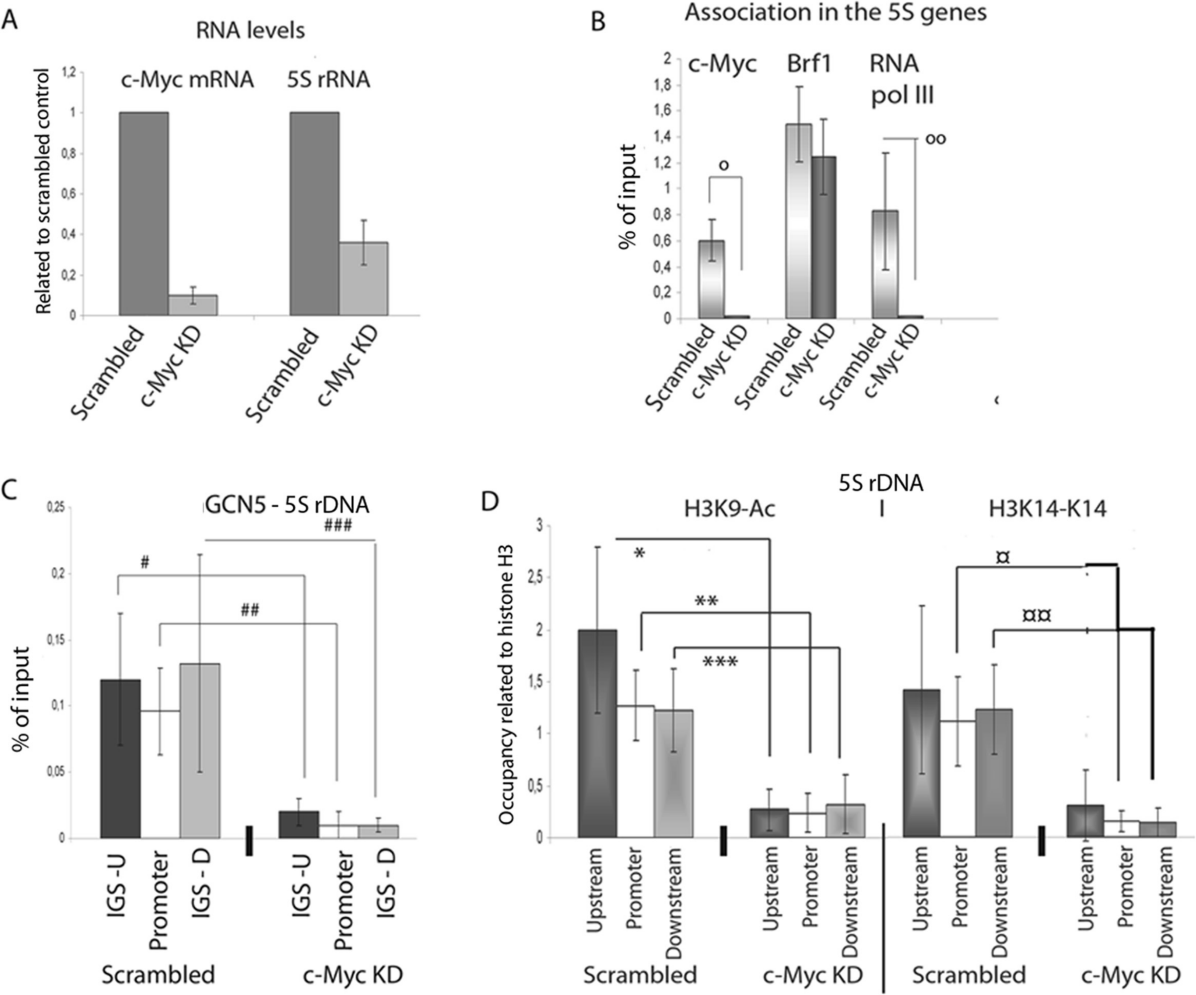
c-Myc recruits HATs, which results in acetylation of H3K9 and H3K14. **(A)** The levels of c-Myc mRNA and 5S rRNA after 30–40 h silencing of c-Myc by siRNA. The error bars (standard deviation.) are from three independent experiments, where the RNA levels were normalised to ARRP mRNA levels and the 18S rRNA levels. **(B)** qPCR analyses of ChIP experiments to show the binding of c-Myc, Brf1 and RNA pol III to the 5S rRNA gene. The error bars represent standard deviations for four independent experiments (o *p* = 0.001; oo *p* = 0.001). **(C)** qPCR analyses of ChIP of GCN5 levels at the 5S rDNA in scrambled and c-Myc KD cells. The error bars represent standard deviations from three independent experiments (# *p* = 0.005; ## *p* = 0.002; ### *p* = 0, 003). **(D)** qPCR of ChIP analyses of H3K9-Ac and H3K14-Ac at the 5S rDNA in scrambled cells and c-Myc KD cells. The values are adjusted to the level of H3 (*p = 0.03; **p = 0.0035; ***p = 0.03; ¤ *p* = 0.003; ¤¤ *p* = 0.0032). The results presented are the means of four independent experiments and the error bars show standard deviations.

To study the function of the association of c-Myc at the 5S rRNA and 7SL genes, we investigated factor binding in c-Myc knock-down cells. Transfection with c-Myc siRNAs for 30 h reduced the level of c-Myc mRNA and the levels of 5S rRNA, 7SL RNA, tRNA, snU6 RNA and the mRNA of cyclin D, but not that of the actin mRNA (Figure 5A and Supplementary Figure S5C). Knock-down of c-Myc not only affected the association pattern of Max at the 5S rDNA and 7SL RNA locus but also affected the binding of other factors. c-Myc knock-down reduced the binding of RNA pol III to the 5S rRNA and 7SL RNA genes, whereas that of Brf1 remained unchanged (Figure 5B and Supplementary Figure S5D). Similarly, Brf1 also remained bound at tRNA genes in c-Myc KD cells, whereas the binding of RNA pol III was reduced (Supplementary Figure S5E). Brf2 is used instead of Brf1 at snU6 genes, and in support of this, we did not detect any Brf1 at the gene. However, RNA pol III was clearly bound to the gene (Supplementary Figure S5E). Taken together, this suggests that c-Myc does not regulate the binding of all RNA pol III factors at the gene. Instead, c-Myc knock-down reduced the binding of hGCN5 at the 5S rDNA and 7SL gene locus (Figure 5C and Supplementary Figure S5F), leading to a reduction of histone H3 acetylation, both H3K9-Ac and H3K14-A (Figure 5D and Supplementary Figure S5G). The level of GCN5 was also reduced at the tRNA genes, snU6 genes and cyclin D (c-Myc regulated RNA pol II gene) (Supplementary Figures S5H and S5J) as well as H3K9-Ac and H3K14-Ac after c-Myc knock-down (Supplementary Figures S5I and S5J), which demonstrates that GCN5 is important for the H3-acetylation and transcription upon c-Myc expression. c-Myc is also responsible for histone H4 acetylation on RNA pol II genes (25,26,40), but we could detect only slight effects on the H4K8-Ac or H4K16-Ac compared to the effect of H3-Ac at the 5S rDNA (Supplementary Figure S6A). The effect of WSTF knock-down rather resulted in an increase in H4K8-Ac and H4K16-A (see Supplementary Figure S6A).

### The c-Myc-Max complex binds in the IGS through DNA and affects 5S rRNA transcription

**Figure 6.**
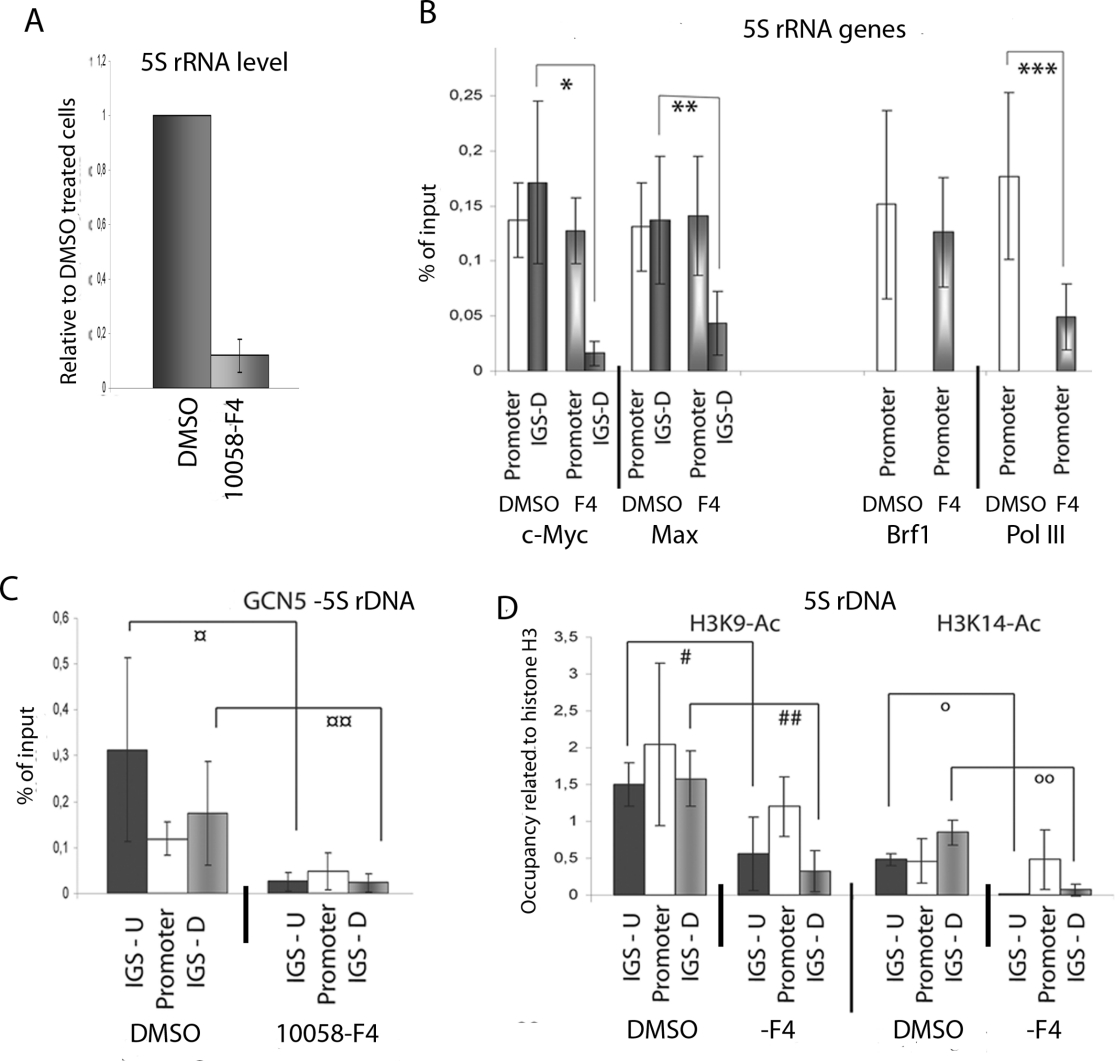
c-Myc binds to DNA as a dimer with Max in the IGS of the 5S rDNA. **(A)** The level of 5S rRNA after 16 h treatment with the inhibitor 10058-F4. The levels are related to the RNA levels in cells treated only with DMSO. The error bars (standard deviation) are from three independent experiments, where the RNA levels were normalised to the 18S rRNA levels. **(B)** The binding of c-Myc, Max, Brf1 and RNA pol III to the promoter and the downstream position (IGS-D) of the5S rDNA in cells treated with 10058-F4 or DMSO assessed by qPCR of ChIP experiments. The error bars (standard deviation) are from four independent experiments. (*p = 0.004; **p = 0.0015; ***p = 0.016). **(C)** qPCR analyses of ChIP experiments to assess the distribution of GCN5 of the 5S rDNA in 10058-F4 treated cells, detected by primer pairs for the upstream (IGS-U), promoter and downstream (IGS-D) regions. The error bars (standard deviation) are from four independent experiments. (¤ *p* = 0.046; ¤¤ *p* = 0.046). **(D)** qPCR analyses of ChIP experiments showing the occupancy of the histone modifications H3K9-Ac and H3K14-Ac at the 5S rDNA in 10058-F4 cells. The values are adjusted to the level of H3. The error bars (standard deviation) are from four independent experiments. (# *p* = 0.009; ## *p* = 0.005; o *p* = 0.009; oo *p* = 0.001).

To investigate whether c-Myc and Max bind directly to DNA in the IGS, we treated cells with the compound 10058-F4, which specifically inhibits the binding between c-Myc and Max, and their binding to E-boxes in DNA (41). Treatment of cells with 75 μM 10058-F4 for 16 h reduced the level of 5S rRNA (Figure 6A). Before determining the binding of factors to chromatin, we established that the protein–protein interaction between c-Myc and Brf1 was not inhibited by 10058-F4 (Supplementary Figure S6B). We treated nuclear extract with 75 μM 10058-F4 for 4 h to examine the effect on the c-Myc interaction to Brf1. This method was used to show the disruption of the binding of c-Myc to Max (42). Next, we tested whether the binding of c-Myc and Max to the 5S rRNA locus was affected by 10058-F4. The compound affected the binding of c-Myc and Max at the gene and at sites in the IGS differently; the binding of both factors at the IGS was reduced, whereas that at the promoter was not affected (Figure 6B). Brf1 remained bound at the gene in cells treated with the compound (Figure 6B), but, despite the presence of both Brf1 and c-Myc at this position, less RNA pol III was bound (Figure 6B). In addition, treatment with 10058-F4 resulted in less GCN5 bound to the 5S rDNA (Figure 6C), and lower H3K9-Ac and H3K14-Ac levels in the IGS (Figure 6D). From these experiments we conclude that c-Myc-Max binding in the IGS is required for histone acetylation, which is, in turn, required for 5S rRNA transcription.

### The binding of c-Myc at the gene affects 5S RNA transcription

**Figure 7.**
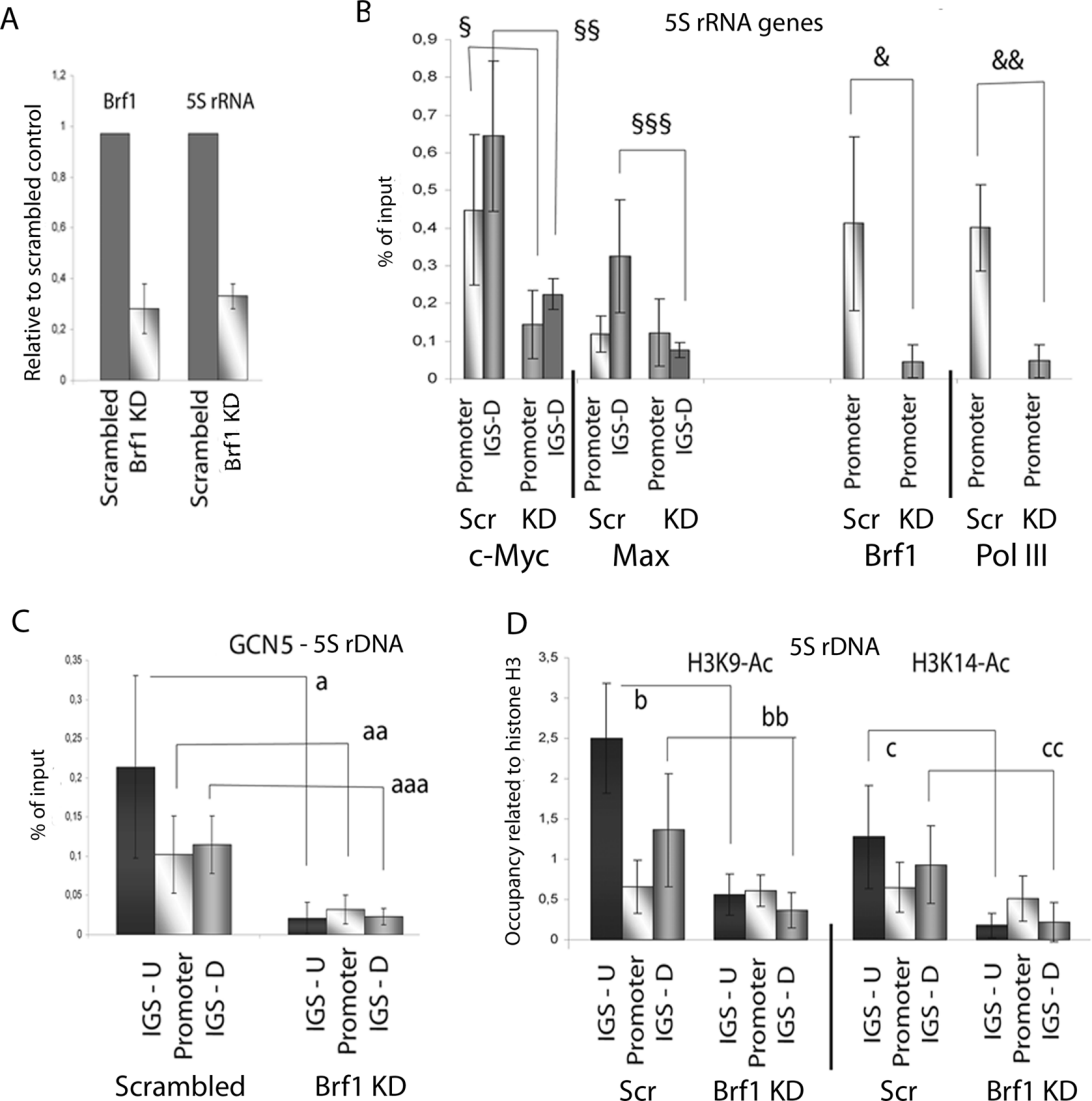
c-Myc binds simultaneously to the promoter and in the IGS. **(A)** The level of 5S rRNA after transfected with Brf1 siRNA for 30 h. The levels are related to the RNA levels in cells transfected with scrambled siRNA. The error bars (standard deviation) are from three independent experiments, where the RNA levels were normalised to the ARRP mRNA and the 18S rRNA levels. **(B)** The binding of c-Myc, Max, Brf1 and RNA pol III to the promoter and the downstream position (IGS-D) of the5S rDNA in cells transfected with scrambled siRNA or Brf1 siRNA for 30 h assessed by qPCR of ChIP experiments. The error bars (standard deviation) are from four independent experiments. (§ *p* = 0.048; §§ *p* = 0.013; §§§ *p* = 0.03). **(C)** qPCR analyses of ChIP experiments to assess the distribution of GCN5 of the 5S rDNA in scrambled and Brf1 KD cells, detected by primer pairs for the upstream (IGS-U), promoter and downstream (IGS-D) regions. The error bars (standard deviation) are from four independent experiments. (a *p* = 0.035; aa *p* = 0.02; aaa *p* = 0.001). The error bars (standard deviation) are from four independent experiments. **(D)** qPCR analyses of ChIP experiments showing the occupancy of the histone modifications H3K9-Ac and H3K14-Ac at the 5S rDNA in scrambled and Brf1 KD cells. The values are adjusted to the level of H3. The error bars (standard deviation) are from four independent experiments. (b *p* = 0.001; bb *p* = 0.009; c *p* = 0.03; cc *p* = 0.007).

Next, we knocked down Brf1 to study the role of the c-Myc site at the gene. The knock-down of Brf1 reduced the levels of 5S rRNA, 7SL RNA and tRNA, but not that of snU6 RNA (Figure 7A and Supplementary Figure S6C). This confirms the findings that Brf1 is required for the transcription of the 5S rRNA genes, 7SL RNA genes and tRNA genes, and not by snU6 genes, which uses Brf2. Knock-down of Brf1 reduced the binding of pol III at the 5S rRNA gene (Figure 7B) in line with the lower level of transcription. The reduction of Brf1 resulted in a decreased binding of c-Myc both the 5S rRNA gene and in the IGS (Figure 7B), whereas the binding of Max was affected only in the IGS. The effect of Brf1 knock-down on the binding of GCN5 and the subsequent acetylation of histone H3 by Brf1 knock-down was similar to that after treatment with the inhibitor 10058-F4; the level of GCN5 was lower in Brf1 KD cells (Figure 7C), as were the levels of H3K9-Ac and H3K14-A outside of the 5S rRNA genes (Figure 7D). Since our results showed that Brf1 knock-down reduces c-Myc binding also at the IGS, we propose that the two sites co-operate to activate transcription. Histone acetylation was retained at the gene in both cells treated with the compound 10058-F4 and in Brf1 KD cells, which could be a result of the internal HAT-activity of the TFIIIC in the RNA polymerase machinery.

### B-WICH acts prior to c-Myc in the regulation of transcription

**Figure 8.**
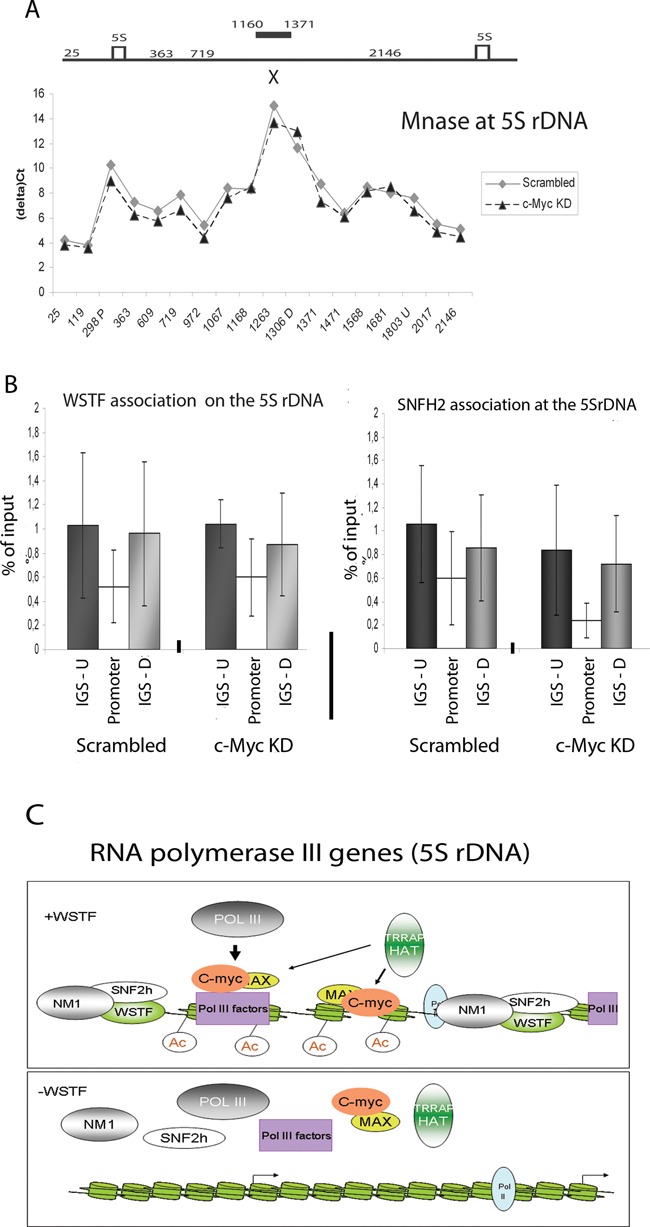
c-Myc requires the B-WICH complex to remodel chromatin for its function. **(A)** MNase accessibility of the 5S rDNA using cross-linked chromatin from scrambled cells and c-Myc KD cells analysed by qPCR using the primer pairs depicted in Figure 2A. The gene organisation is depicted above the graph, where the hypersensitive site affected by B-WICH is marked by a bar and the E-box by an X. The graph is based on four independent experiments. **(B)** qPCR analyses of ChIP experiments of the distribution of the WSTF and SNF2h in scrambled and c-Myc KD cells at the 5S rDNA. The position detected by the primer pairs are marked IGS-U for upstream, promoter for the internal promoter in the gene and IGS-D for downstream. The results are based on four independent experiments and the error bars represent standard deviations. **(C)** Schematic model of the function of the B-WICH complex in RNA pol III transcription, here depicted at the 5S rDNA.

WSTF knock-down had a greater effect on the factor binding at the 5S rDNA locus than c-Myc or Max knock-down. This led us to investigate the order of events between WSTF and c-Myc in transcriptional activation. To determine whether c-Myc knock-down and the subsequent reduction of histone H3 acetylation lead to a similar remodelling of the chromatin structure as that observed in WSTF KD cells, we performed high-resolution MNase walking. In contrast to WSTF knock-down (Figure 2C), c-Myc knock-down did not result in a changed chromatin structure at the 5S rDNA (Figure 8A). This clearly demonstrates that the B-WICH acts at a step prior to c-Myc. This is further supported by the finding that the binding of the B-WICH complex components WSTF and SNF2h was not compromised in c-Myc KD cells (Figure 8B). WSTF and SNF2h were also present at the 5S rDNA locus in cells treated with the compound 10058-F4 and in Brf1 KD cells (Supplementary Figure S6D). We conclude that the B-WICH complex acts prior to c-Myc in the activation of 5S rRNA transcription. We propose a model in which B-WICH is required for the remodelling of chromatin such that a site, most likely an E-box at the 5S rDNA, becomes accessible for the c-Myc-Max complex (Figure 8C). The c-Myc-Max complex subsequently recruits further factors, such as HATs, to obtain the H3 acetylation that is necessary for transcriptional activation.

## DISCUSSION

This study demonstrates that chromatin is an important regulator of RNA pol III transcription and that chromatin-remodelling complexes are required to alter the chromatin structure in the vicinity of the genes for transcriptional activation. We have studied the order of events upon transcriptional activation of 5S rRNA and 7SL RNA genes and demonstrated that chromatin remodelling is required early in the process. We demonstrate that the B-WICH complex is necessary for the remodelling of chromatin in the vicinity of the 5S and 7SL to allow the binding of other factors that induce RNA pol III transcription (Figure 8C). The site exposed by the B-WICH complex in the IGS of the 5S rDNA contained a novel c-Myc-Max DNA binding site and we propose that c-Myc regulates 5S rRNA transcription by binding to two sites; one site via Brf1 at the gene promoter, and one c-Myc-Max DNA binding site in the IGS.

It has previously been proposed that transcription factors affect the chromatin structure and the profile of histone modifications in the vicinity of RNA pol III genes. These factors mainly prevent nucleosomes from forming at the promoters of the genes. One example is TFIIIC, which remains bound at low levels in the absence of transcription (13,19,28,43). Genome-wide studies of the global state of RNA pol III genes have shown that active RNA pol III genes have histone modifications that resemble those of RNA pol II genes (18–20,37,38). It is unclear how these modification states are set, but it has been proposed that RNA pol II transcription factors, such as c-Myc, are involved (37). Chromatin-remodelling complexes are also associated with RNA pol III transcription; RSC in yeast regulates the nucleosome density and positioning at tRNA genes (28,29). We propose that B-WICH acts by a similar mechanism, positioning nucleosomes or altering their structure, not only at the genes but also at a distance. WSTF knock-down results in a global heterochromatinisation (44), and we here present findings that B-WICH also acts locally on ribosomal genes. WSTF is part of a further complex, the WICH complex, which is involved in spacing of nuclesomes during replication (45). The global heterochromatinisation is most likely caused by a deficiency in WICH activity. However, we have shown that WSTF is also involved in transcription (32,33) by locally regulate the chromatin structure at the RNA pol I gene (35). Here, we demonstrate that also the alteration of the chromatin structure at the 5S rDNA and at the 7SL RNA locus depends on WSTF. This suggests that the WSTF protein together with SNF2h has dual functions; in replication/DNA repair and in transcription of a subset of genes, such as ribosomal genes. We propose that the role in transcription is to remodel chromatin. The change in the chromatin structure at the 5S rDNA and 7SL RNA locus induced by B-WICH makes the DNA more accessible to transcription factors and is a prerequisite for factor loading at these genes and for the activation of transcription.

We have identified c-Myc as one factor that requires B-WICH to remodel chromatin at the 5S rDNA. c-Myc binding was also B-WICH-dependent at the 7SL RNA genes, but RNA pol II is also a factor that depends on B-WICH for binding. This suggests that B-WICH is not specific to any one factor, but is used to activate genes by remodelling chromatin at specific sites. Previous studies have shown that c-Myc binds to RNA pol III genes via Brf1 in the TFIIIB complex in a Max-independent manner (14–16). We show here that c-Myc associates with the 5S rDNA at two positions, one with Brf1 in TFIIIB at the promoter and one, most likely via an E-box, in the IGS. The binding in the IGS was sensitive to treatment with the compound 10058-F4, which interferes with the DNA-binding of the c-Myc-Max dimer (41). This demonstrates that the site in the IGS is a conventional c-Myc-Max DNA-binding site. Max homeodimers, and Mxd1-Max heterodimers also bind to E-boxes, but we detected less Max in the IGS in c-Myc KD cells, Brf1 KD cells, and cells treated with the compound 10058-F4. The binding pattern of Max dimers is further influenced by both post-translational modifications and acetylated and phosphorylated Max-homeodimers bind less efficiently to E-boxes (46,47). The binding of c-Myc at the promoter was not affected by 10058-F4, suggesting a protein–protein interaction via Brf1. Max also bound the promoter in a 10048-F4-independent way, suggesting that also Max bind to the RNA machinery by protein–protein interactions. Max also bound to the promoter in Brf1 KD cells, which suggests an interaction with another component. We have, however, not elucidated the nature of this interaction, as IPs performed at 0.42 M KCl nuclear extract with antibodies against components of the RNA pol III transcription machinery have not revealed any binding partners.

We suggest that the association of c-Myc to both a DNA binding site, probably in the IGS, and the binding site in the promoter is required for transcription. These sites were both required for the recruitment of HATs and the subsequent histone acetylation of the locus. Since interfering with binding of c-Myc at the promoter affected the binding of c-Myc-Max at the IGS, we suggest that the two sites in the repeat co-operate. It has been reported that c-Myc acts as a linker forming functional chromatin loops at the nucleolar rDNA loci (48). Thus, similar functional loops may exist in 5S rDNA, since the two c-Myc-Max binding sites in the 5S rDNA influence each other and show similar responses.

The identification of a novel c-Myc-Max site links for the first time 5S rRNA transcription to the Myc/Max/Mxd gene regulatory network, and this may explain the deregulation of RNA pol III transcription often seen in oncogenic transformation (49). However, the B-WICH is not specific for c-Myc, as it is required for the binding of both c-Myc and RNA pol II around the 7SL RNA genes. Thus, the function of B-WICH may be to prepare the chromatin in the vicinity of target genes. The MNase digestions performed on WSTF knock-down and c-Myc knock-down chromatin showed a difference in DNA accessibility only in WSTF knock-down cells, a finding which supports this conclusion. MNase digests linker DNA (50), and the results presented here suggest that B-WICH is required to move nucleosomes or proteins to make certain region less compact. The decreased level of acetylation upon c-Myc knock-down did not affect the accessibility of linker DNA. This presents an order of event with the B-WICH complex acting prior to c-Myc-Max binding. It is possible that different chromatin remodelling complexes operate at different classes of RNA pol III genes, as DNA-methylation patterns affect RB binding at the snU6 genes (51). We propose that B-WICH acts specifically to activate the transcription of the two RNA pol III genes; the 5S rRNA and the 7SL RNA genes.

## SUPPLEMENTARY DATA

Supplementary Data are available at NAR Online.

SUPPLEMENTARY DATA
