# Supplementary material for: The B-WICH chromatin-remodelling complex regulates RNA polymerase III transcription by promoting Max-dependent c-Myc binding
Source: Nucleic Acids Res. 2015 Apr 16;43(9):4477–90. doi: 10.1093/nar/gkv312 (PMC4482074; doi:10.1093/nar/gkv312)
Supplement: SUPPLEMENTARY DATA [file supp_43_9_4477__index.html]

The B-WICH chromatin-remodelling complex regulates RNA polymerase III transcription by promoting Max-dependent c-Myc binding — SUPPLEMENTARY DATA 

# The B-WICH chromatin-remodelling complex regulates RNA polymerase III transcription by promoting Max-dependent c-Myc binding

## SUPPLEMENTARY DATA

**Files in this Data Supplement:**

- SUPPLEMENTARY DATA
