# Supplementary material for: The B-WICH chromatin-remodelling complex regulates RNA polymerase III transcription by promoting Max-dependent c-Myc binding
Source: Nucleic Acids Res. 2015 Apr 16;43(9):4477–90. doi: 10.1093/nar/gkv312 (PMC4482074; doi:10.1093/nar/gkv312)
Supplement: SUPPLEMENTARY DATA [file supp_gkv312_nar-03526-v-2014-File010.pdf]

Fatemeh Sadeghifar, Stefanie Böhm, Anna Vintermist and Ann-Kristin Östlund Farrants

The B-WICH chromatin-remodelling complex initiates the regulation of RNA polymerase III by promoting the binding of c-Myc

Supplemental Figures:

Figure S1

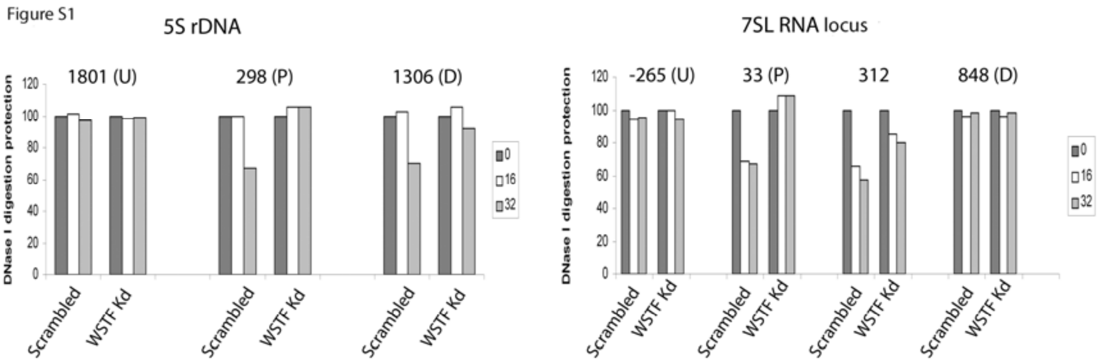

Figure S1: WSTF knock down leads to a more protected DNA at the promoter and downstream of the 5S rRNA gene and around the 7SL genes. DNase I digestion (Material and Methods below) of WSTF KD cells and cell transfected with scrambled siRNA for the times indicated. DNA was extracted and regions at the 5S rDNA and the 7SL genes were amplified, using the primer pair giving products at the positions indicated above the bars. The positions are depicted in Figure 2A, U is upstream, P is the internal promoter, D is downstream. The signal intensities of the products were qualified using ImageQuant, Bio-RAD, n=3.

Materials and Method: Cells were treated with LPC-buffer (0.01% L-α-lysophosphatidylcholine, 150 mM sucrose, 80 mM KCl, 35 mM Hepes at pH 7.4, 5 mM K_2HPO_4, 5 mM $MgCl_2$, 0.5 mM $CaCl_2$) for 90 seconds. Cells were treated with 1 U DNaseI in DNase-buffer (20 mM Tris-HCl at pH 7.5, 60 mM KCl, 15 mM NaCl, 250 mM sucrose, 1mM $CaCl_2$, 1mM DTT and protease inhibitors) for the times indicated, then stopped by adding 3.3 mM EDTA. The samples were treated with proteinase K, 1% SDS was added, and the DNA was extracted with phenol:chloroform. The samples were digested with 10 U of EcoRI and 10 U of HindIII, and were analysed by PCR over the region indicated.

Figure S2

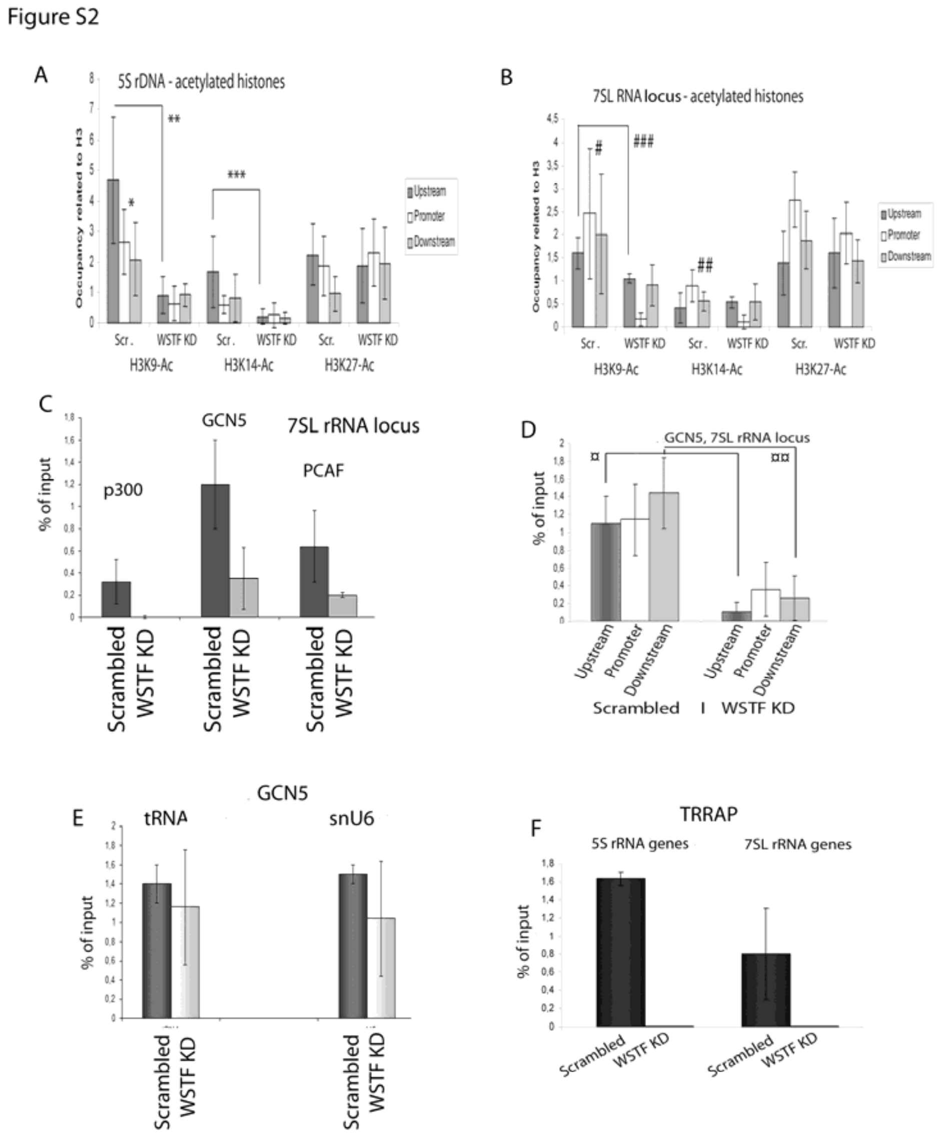

(A) The distribution of H3K9-Ac, H3K14-Ac, and H3K27-Ac in scrambled cells (Scr) and WSTF KD at the upstream, promoter and downstream position at the 5S rDNA. (***p*= 0.006; ****p*= 0.0046, Student's t-test). The results presented are the means of five independent experiments, and the signals are presented as Occupancy related to H3. The error bars show standard deviations, * is the p for the reduced binding at the gene presented in Figure 3B. **(B)** The distribution of H3K9-Ac, H3K14-Ac, H3K27-Ac in scrambled cells (Scr) and WSTF KD upstream, promoter and downstream of the 7SL gene locus (### *p*= 0.0046, Student's t-test,). The results presented are the means of five independent experiments, and the signals are presented as Occupancy related to H3. The error bars show standard deviations, # and ## is the p for the reduction in binding at the gene presented in Fig 3C. **(C)** qPCR of ChIP analyses of the association of p300, GCN5

and PCAF at the 7SL RNA gene of scrambled cells and WSTF KD cells. The results presented are the means of three independent experiments, and the signals are presented as percentage of input signal. The error bars show standard deviations. **(D)** qPCR of ChIP analyses in cells transfected with scrambled siRNA (Scrambled) and cells transfected with WSTF siRNA (WSTF KD). The different position of the 7SL RNA locus were detected using primer pairs for the upstream, promoter and downstream regions (# p=0.0037; ## p=0.027, Student's t-test). The results presented are the means of four independent experiments, and the signals are presented as percentage of input signal. The error bars show standard deviations. **(E)** qPCR of ChIP analyses of the GCN5 to the tRNA and snU6 genes in scrambled cells transfected with scrambled SiRNA (Scrambled) and cells transfected with WSTF siRNA (WSTF KD). The results presented are the means of three independent experiments, and the signals are presented as percentage of input signal. The error bars show standard deviations. **(F)** qPCR of a ChIP analysis of the TRRAP protein at the internal promoter of the 5S rRNA and the 7SL RNA gene in cells transfected with scrambled siRNA (Scrambled) and cells transfected with WSTF siRNA (WSTF KD). The results presented are the means of three independent experiments, and the signals are presented as percentage of input signal. The error bars show standard deviations.

Fig S3

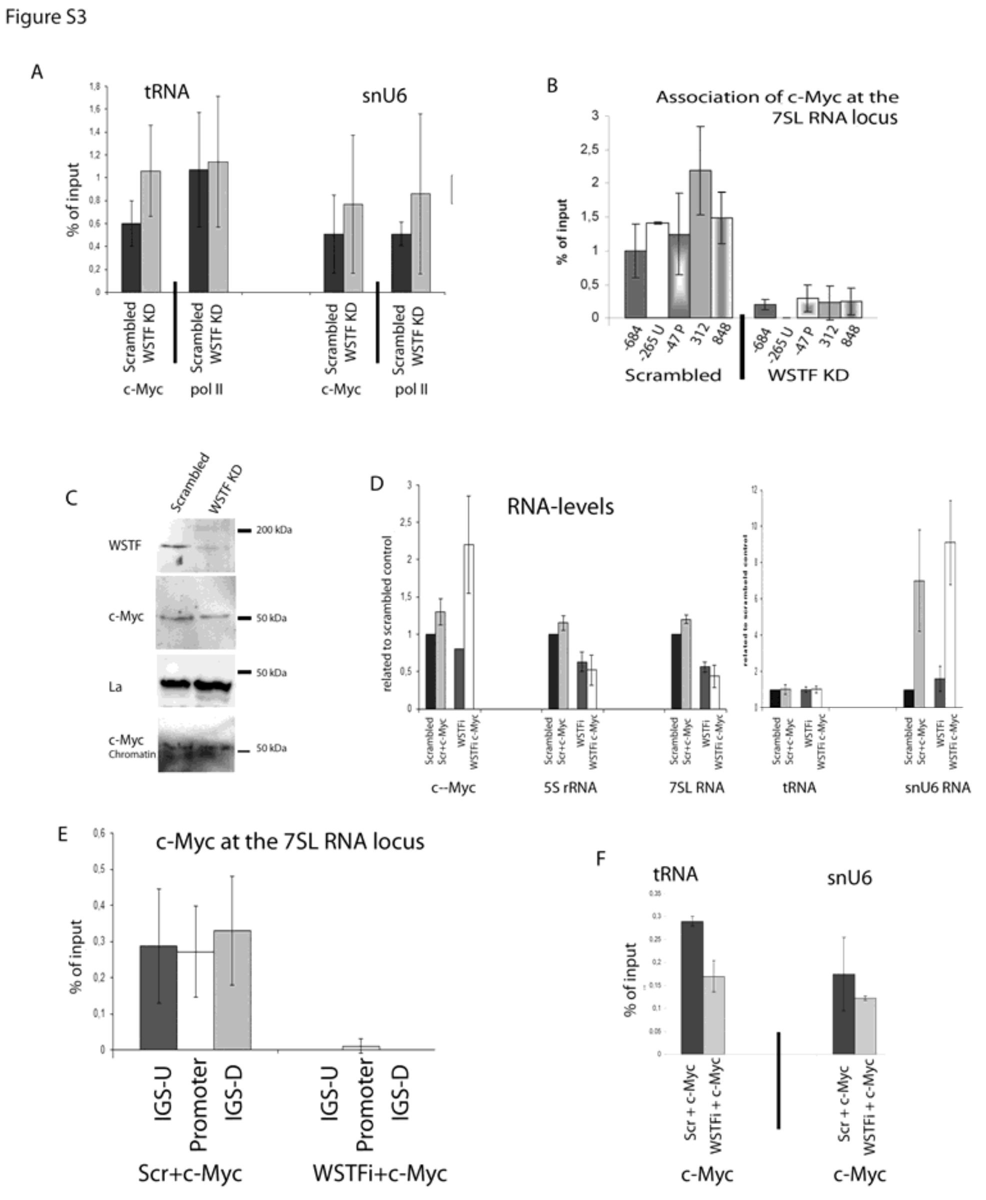

A) qPCR of ChIP analyses of the c-Myc and RNA pol II to the tRNA and snU6 genes in scrambled cells transfected with scrambled siRNA (Scrambled) and cells transfected with WSTF siRNA (WSTF KD). The results presented are the means of four independent experiments, and the signals are presented as percentage of input signal. The error bars show standard deviations.**B)** Distribution of the association of c-Myc at the 7SL RNA locus by ChIP in scrambled and WSTF KD cells. The numbers of the primer products refer to the no depicted in Figure 2C. The error bars represent standard deviations of four independent experiments. It has been suggested that c-Myc associate strongest approximately 200 bp upstream of RNA pol genes, which corresponds to a position at

265 upstream in the 7SL RNA locus. **C)** The protein level of c-Myc is reduced upon WSTF knock down. Immunoblotting of a HeLa cells nuclear extracts (0.7 M NaCl) and the proteins subsequently separated on a 10% SDS polyacrylamide gel electrophoresis. The antibodies used to detect interacting proteins are shown on the left. The molecular weights are given to the right. The La protein was used as loading control. c-Myc chromatin denotes the level of protein detected in the chromatin pellet after brief fractionation of nuclei using 0.4 M NaCl to extract proteins. **D)** RNA levels in cells transfected with scrambled siRNA, scrambled siRNA plus an expression vector for c-Myc, WSTF siRNA, or WSTF siRNA plus an expression vector for c-Myc. The RNA levels were normalised to 18S. **E)** The occupancy of c-Myc at the internal promoter of the 7SL RNA gene and in the IGS-D in cells in which WSTF has been knocked down and c-Myc expressed exogenously compared to cells transfected with scrambled siRNA and overexpressing c-Myc. The error bars (standard deviation) are from four independent experiments. **F)** The occupancy of c-Myc at tRNA and snU6 genes in cells in which WSTF has been knocked down and c-Myc expressed exogenously compared to cells transfected with scrambled siRNA and overexpressing c-Myc. The error bars (standard deviation) are from four independent experiments.

Figure S4

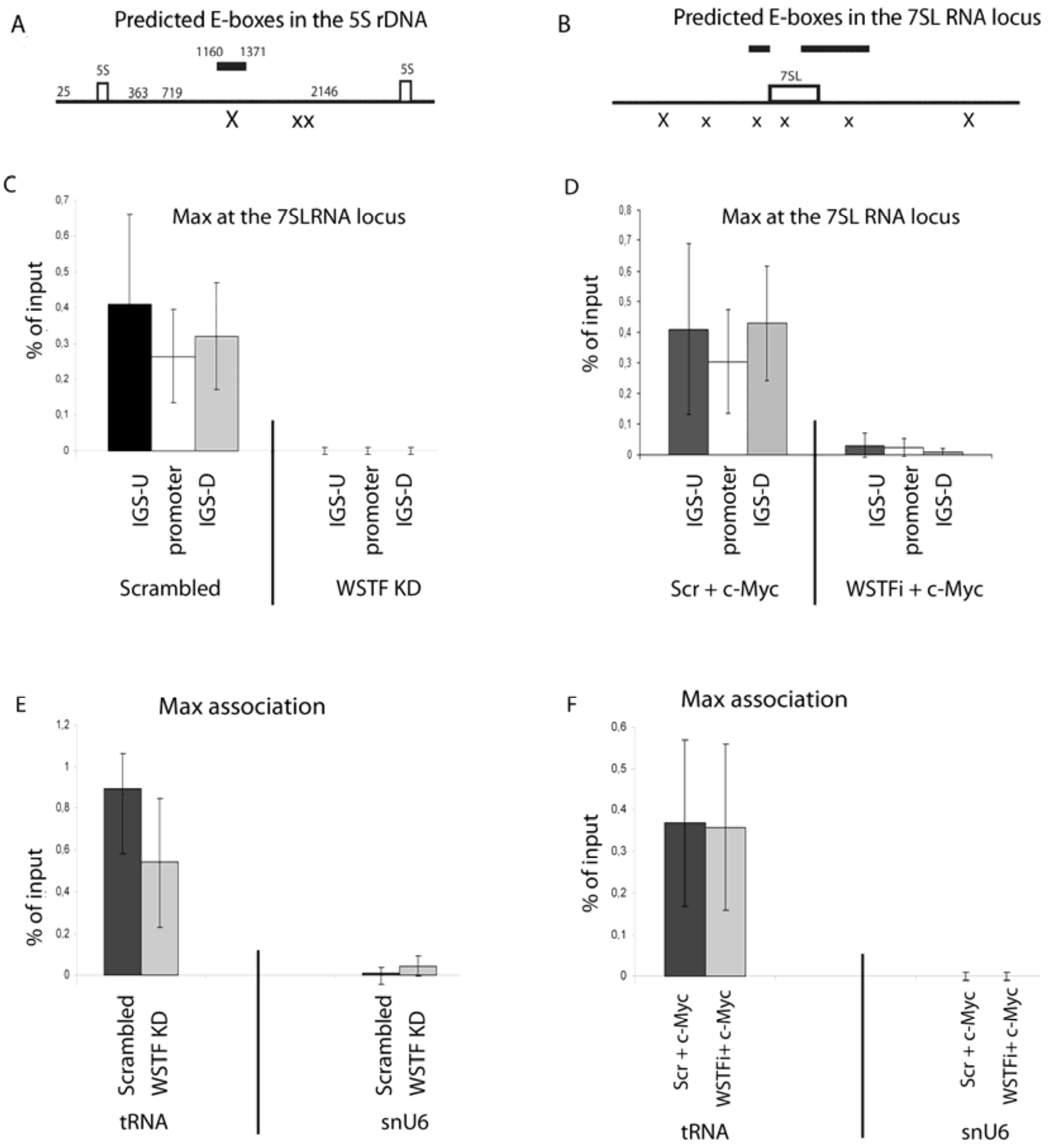

A) E-boxes are present in the 5S rDNA and the 7SL RNA genes. A) The 5S rRNA gene repeat is depicted and shows the positions of E-boxes, where X denotes canonical E-boxes and x denotes non-canonical E-boxes. The positions are referred to by numbers of the 5S rDNA locus (accession no X12811.1). The position affected by B-WICH activity is marked with a bar above the gene repeat. **B)** The 7SL RNA locus shows the positions of E-boxes, where X denotes canonical E-boxes and x denotes non-canonical E-boxes. The position affected by B-WICH activity is marked with bars above the gene repeat. **C)** Max binds to the 7SL gene locus in a WSTF dependent manner. Distribution of the association of Max at the 7SL DNA locus by ChIP from chromatin from scrambled and WSTF KD cells. The primer pairs detect the upstream (IGS-U), the promoter and the

downstream (IGS-D) positions. The error bars (standard deviation) are from three independent experiments. **D)** The occupancy of Max at the internal promoter of the 7SL RNA locus; at the IGS-U, the promoter, and IGS-D in cells in which WSTF has been knocked down and c-Myc expressed exogenously compared to cells transfected with scrambled siRNA and overexpressing c-Myc. The error bars (standard deviation) are from three independent experiments. **E)** The occupancy of Max at the tRNA and snU6 genes in cells in which WSTF has been knocked down with siRNA compared to cells transfected with scrambled siRNA. The error bars (standard deviation) are from three independent experiments. **F**) The occupancy of Max at the tRNA and snU6 RNA genes in cells in which WSTF has been knocked down and c-Myc expressed exogenously compared to cells transfected with scrambled siRNA and overexpressing c-Myc. The error bars (standard deviation) are from three independent experiments. The effect on Max binding to the tRNA was not affected by WSTF knockdown even in cells where c-Myc is expressed. c-Myc expression could not increase the binding of Max to snU6 RNA genes, and it was even under these conditions lacking.

Figure S5

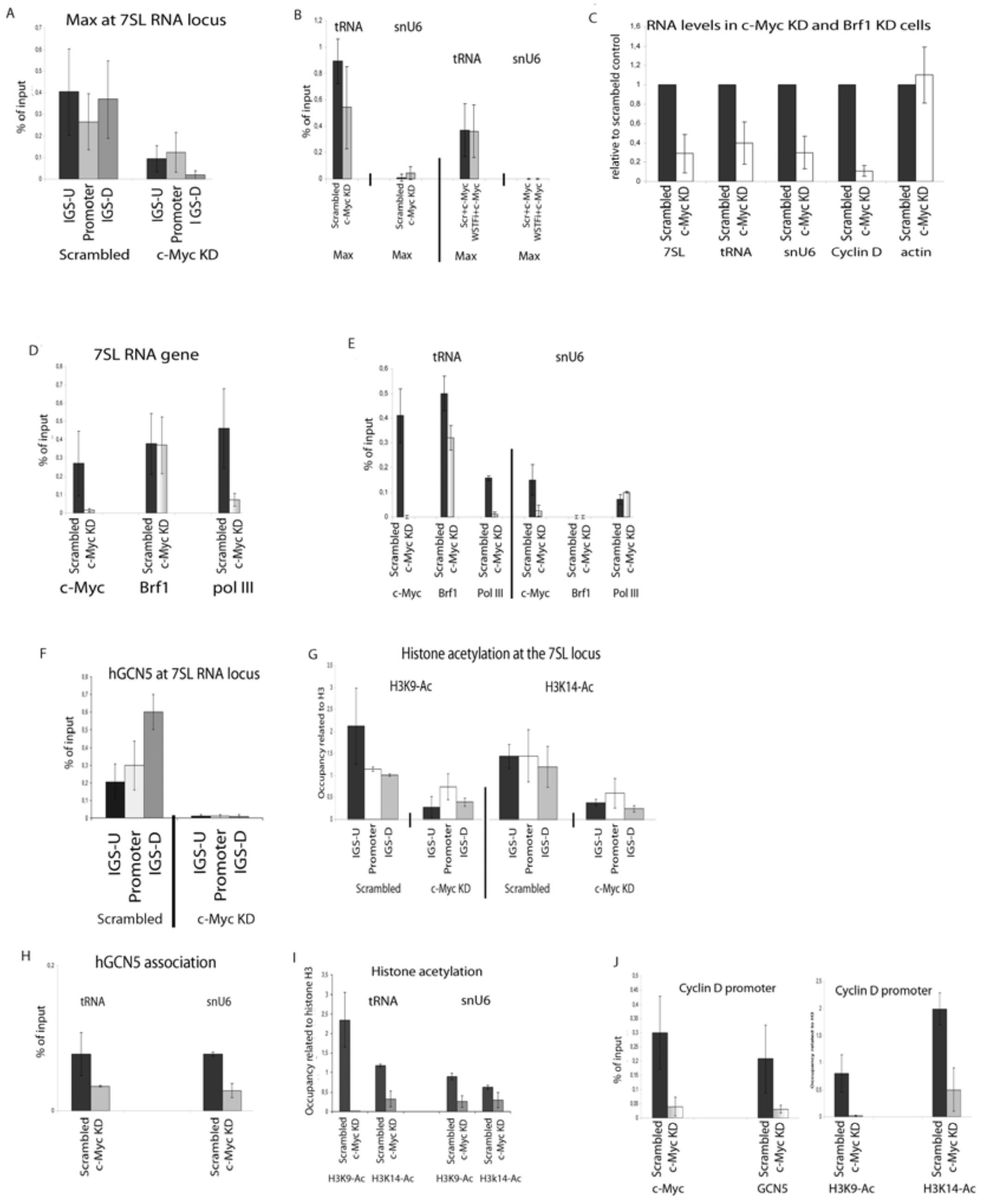

c-Myc know down reduces the binding of RNA pol III, GCN5 and histone acetylation at the 7SL rRNA locus and tRNA, whereas GCN5 and histone H3 acetylation is reduced without a decreased binding of RNA pol III. A) qPCR analyses of ChIP experiments to show the binding of Max to the 7SL RNA locus in cells transfected with scrambled siRNA or c-Myc siRNAs. The error bars represent standard deviations for four independent experiments. **B)** qPCR analyses of ChIP experiments to show the binding of Max to tRNA and snU6 RNA genes in cells transfected with

scrambled siRNA or c-Myc si RNAs. The error bars represent standard deviations for three independent experiments. **C)** The levels of 7SL RNA, tRNA, snU6 RNA, cyclin D mRNA, and actin mRNA after 30 hours silencing of c-Myc by siRNA. The error bars (standard deviation) are from three independent experiments, where the RNA levels were normalised to the 18S rRNA levels. The levels are presented as related to that in scrambled control. **D)** qPCR analyses of ChIP experiments to show the binding of c-Myc, Brf1 and RNA pol III to the 7SL RNA gene in cells transfected with scrambled siRNA or c-Myc siRNAs. The error bars represent standard deviations for four independent experiments. **E)** qPCR analyses of ChIP experiments to show the binding of c-Myc, Brf1 and RNA pol III to the tRNA and snU6 genes in cells transfected with scrambled siRNA or c-Myc siRNAs. The error bars represent standard deviations for four independent experiments. **F)** qPCR analyses of ChIP of GCN5 levels at the 7SL rRNA locus in scrambled and c-Myc KD cells. The error bars represent standard deviations from three independent experiments. **G)** qPCR of ChIP analyses of H3K9-Ac and H3K14-Ac at the 7SL RNA locus in scrambled cells and c-Myc KD cells. The values are adjusted to the level of H3. The results presented are the means of four independent experiments and the error bars show standard deviations. **H)** qPCR analyses of ChIP of GCN5 levels at tRNA and snU6 RNA genes in scrambled and c-Myc KD cells. The error bars represent standard deviations from three independent experiments. **I)** qPCR of ChIP analyses of H3K9-Ac and H3K14-Ac at the tRNA and snU6 RNA genes in scrambled cells and c-Myc KD cells. The values are adjusted to the level of H3. The results presented are the means of four independent experiments and the error bars show standard deviations. **J**) qPCR of ChIP analyses of c-Myc, GCN5, H3K9-Ac and H3K14-Ac at the cyclin D promoter in scrambled cells and c-Myc KD cells. The values for c-Myc and GCN5 are related to percent of input and the values for H3K9-Ac and H3K14-Ac are adjusted to the level of H3. The results presented are the means of three independent experiments and the error bars show standard deviations. No binding of Brf1 and RNA pol III could be detected at the cyclin D promoter (not shown).

Figure S6

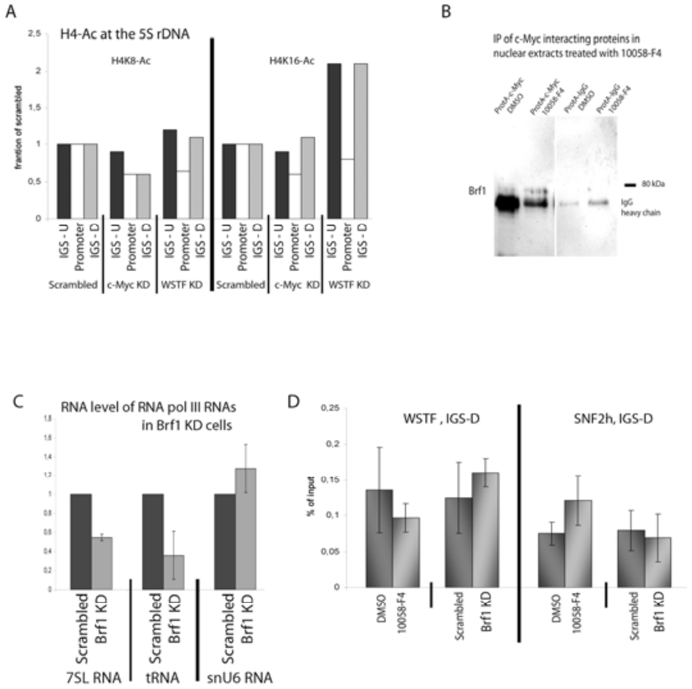

knock down of Brf1 or treatment with the compound 10058-F4 does not affect the binding of WSTF and SNF2h to the 5S rDNA.
A) H4K8-Ac and H4K16-Ac are not affected to the same extent as the H3K9-Ac and H3K13-Ac by c-Myc knock down or WSTF knock down**.** qPCR of ChIP analyses determining the occupancy of histone H4 acetylation; H4K8-Ac and H4K16-Ac at the internal promoter of the 5S rRNA gene in cells transfected with scrambled siRNA, c-Myc siRNA or WSTF siRNA. The values are adjusted to the level of H4 and related to the levels in scrambled controls at each position. The largest effect was observed on the H4K16-Ac in WSTF KD cells, in which the levels in the IGS increased. c-Myc knock down had no great effect on the H4-acetylation level at the 5S rDNA locus. **B)** The compound 10058-F4 does not break the interaction between c-Myc and the TFIIIB component Brf1. Immunoblot of an IP with c-Myc antibodies in nuclear extract (0.4 M NaCl) treated with 75 μM 10058-F4 or an equivalent volume of DMSO for 4 hours. The precipitated proteins were then separated on a 10% SDS-PAGE, transferred to a PVDF membrane and probed with Brf1 antibodies. The molecular weight marker is depicted on the right, as is the position of the IgG heavy chain. The position of Brf1 is marked to the left. A Brf1 signal is seen both in untreated and 10058-F4 treated cells. IgG was used as a negative control for precipitated proteins in both untreated nuclear extracts and in extracts treated with 10058-F4. **C)** The levels of 7SL RNA, tRNA and snU6 RNA after 30 hours silencing of c-Myc by siRNA. The error bars (standard deviation) are from three independent experiments, where the RNA levels were normalised to the 18S rRNA level**s**. The levels are presented as related to that in scrambled control. **D)** The associations of WSTF and SNF2h to the 5S rDNA are not affected by treatment of the

compound 10058-F4 or by Brf knockdown. The association of WSTF and SNF2h at the downstream (IGS-D) position of the 5S rDNA in cells treated with 75 μM 10058-F4 for 16 hours (or with equivalent volume of DMSO) or transfected with scrambled siRNA or with Brf1 siRNA. The results presented are the means of four independent experiments, and the signals are presented as percentage of input signal. The error bars show standard deviations.

Supplemental Information – Table S1

Table S1

SiRNA and c-Myc primers for mRNA analyses

SiWSTF: GGAAGGAGAGAGAGUAUUATT

Si-c-Myc1: CAUCAUCAUCCAGGAGUGUAUTT
Si-c-Myc2: CGAGGUAAAACGGAGCUUUTT

SiBrf1: AAGCACUGSCCCACUUAUUUGTT

SiMax1: GGAGUGAGUGAGUGAGUGATT
SiMax2: CUC AGU CCC AUC ACUCCAATT

Siscrambled: GUGCGAGGGGGUUGUAAUCTT

C-Myc primers for cDNA analysis

Forward 1: CCAGACCTCGAGTTTGAC
Reverse1: GTTCTCAAGGAGCTC

Forward 2: TGGACAGTGTCAGAGCCTG
Reverse 2: CCTTTTCATTGTTTTCCAC

Brf1 primers for cDNA analysis
Forward: GAGGTCATCAGTGTGGTCAA
Reverse: TGGATATTTCACCTTCAACC

Supplemental Information – Table S2

Table S2

Primer pairs 5S rDNA
(Numbers refer to no in gene locus accession no X12811.1)

Forwards	Reverse
25f: 5'-CAGGGCGGAGGACCGGA-3'	25r: 5'-GAGCTTCCACCACATCGG-3'
119f: 5'-CTCGGGAGCGCGGGAG-3'	119r: 5'-CGGATTGCAGCCGACACC-3'
**298 Uf: 5'-CCCGATCTCGTCTGATCTC-3**	**298 Ur: 5'-CGGAGCTTGCAGTGAGCC-3**'
363f: 5'-CGCCTCCCGGGTTCAC-3'	363r:5'-GGTGAAAGCCCGTCTCTAG-3'
609f: 5'-CCTGTTAGCCGGGATGGTC-3'	609r: 5'-GCCTACAGCAGGCCGG-3'
719f: 5'-GCTTTTGTGGCTTCCCCGC-3'	719r: 3'-GACCCCCAGCAGGAGGA-3'
972f: 5'-CTGGGTTCCCTGCCGTTC-3'	972r: 5'-GGTTTGGAAGGGTGCGACG-3'
1097f: 5'-GGGGAAGGGGCGGGCA-3'	1067r: 5'-GGCGAAGCAATGTCCTTCC-3'
1168f: 5'-CCAGCCACCGGGAAAACAG-3'	1168r: 5'-GAGGCCTGGGTCTCTGG-3'
1263f: 5'-AGGCCTCCGGGCCCG-3'	1263; 5'-GCCTTTCTTGGCACCTCC-3'
1**306 Df: 5'-CGCGCTCTGCAAATCTC-3'**	**1306 Dr: 5'-GAGCGAGAGCGATAGAGCC-3'**
1371f: 5'-CAGGAGCCTACGAAACCCAC-3'	1371r: 5'-CAGAACACGGGGGCCAG-3'
1471f: 5'-GTTCTGGCGCAGCCCAAG-3'	1471r: 5'-CGAGAGCGATAGAGCCTTAG-3'
1565f: 5'-CGCTCTCTAGCTCCCTCC-3'	1565r: 5'-GCTGAGAAAGAGAGAGGCACAG-3'
1568f: 5'-CTCGCTCTCTAGCTCCCTC-3'	1568r: 5'-GCACAGAGAAAGAGAGAGAG-3'
1681f: 5'-GTGCCTCTCTCTTTCTCAGC-3'	1681r: 5'-GAGAGCAAGGACGAGATGGAT-3'
**1805 Pf: 5'-CCTTCAAGCCGCGTGTGTG-3'**	**1805 Pr: 5'-GCACTAGAGGCGGCGGC-3'**
2017f: 5'-CCAGCCCGGGGCAAAACA-3'	2017r: 5'-CGGGCCTTTCTCTCACAAC-3'
2146f: 5'-TGGGCCGGCTGTTCGCCT-3'	2146r: 5'-CTGTCGCGGAGGCAGCGTT-3'

Forwards primer and reverse primer sequences are given, with the primer pairs used as upstream (U), promoter (P) and downstream (D) in bold.

Supplemental Information – Table S3

Table S3

Primer pairs 7SL RNA gene
(Numbers refer to bp from transcription start site of gene 7SL RNA 1)

Forwards	Reverse
-684f: 5'-GCTCTTGGTCGAATGCTCAG-3'	-684r: 5'-GCAGGCGTACTCCATGTTG-3'
-463f: 5'-CGACTGTAAGAAAAGAGACAGC-3'	-463r: 5'-TACTGTGGCTTGGGAGTCC-3'
**-265 Uf: 5'-GGTGGCTCCAGTACAGCTGC-3'**	**-265Ur: 5'-CCCAGCGAAACAACCTGACC-3'**
-47f: 5'-GGTCAGGTTGTTTCGCTGG-3'	-47r: 5'-CGAGTAGCTGGGACTACAGG-3'
**+33 Pf: 5'-CTACTCGGGAGGCTGAGG-3'**	**+33Pr: 5'-GAGGTCACCATATTGATGCCG3'**
+203f: 5'- ATGGTGACCTCCCGGGAG-3'	+203r: 5'-GCGATCCCACTACTGATCAG-3'
+312f: 5'-GCGCCTGTGAATAGCCACT-3'	+312r: 5'-CTCCTTTCCCAAGTGTAACG-3'
**+848 Df: 5'-GATACCTACATTCCATATGGTC-3'**	**+848 Dr: 5'-CTCTGACTCCCCTCCTTCC-3'**
+1084f: 5'-GGAGGGGAGTCAGAGATTTG-3'	+1084r: 5'-GCTGTACATATGTTGTTGGTGC-3'

Forwards primer and reverse primer sequences are given, with the primer pairs used as upstream (U), promoter (P) and downstream (D) in bold.
